# Fine-mapping of the human leukocyte antigen locus as a risk factor for Alzheimer disease: A case–control study

**DOI:** 10.1371/journal.pmed.1002272

**Published:** 2017-03-28

**Authors:** Natasha Z. R. Steele, Jessie S. Carr, Luke W. Bonham, Ethan G. Geier, Vincent Damotte, Zachary A. Miller, Rahul S. Desikan, Kevin L. Boehme, Shubhabrata Mukherjee, Paul K. Crane, John S. K. Kauwe, Joel H. Kramer, Bruce L. Miller, Giovanni Coppola, Jill A. Hollenbach, Yadong Huang, Jennifer S. Yokoyama

**Affiliations:** 1 Department of Neurology, University of California, San Francisco, San Francisco, California, United States of America; 2 University of Washington School of Medicine, Seattle, Washington, United States of America; 3 Gladstone Institute of Neurological Disease, San Francisco, California, United States of America; 4 Johns Hopkins University School of Medicine, Baltimore, Maryland, United States of America; 5 Department of Radiology and Biomedical Imaging, University of California, San Francisco, San Francisco, California, United States of America; 6 Brigham Young University, Provo, Utah, United States of America; 7 Departments of Neurology and Psychiatry, Semel Institute for Neuroscience and Human Behavior, University of California, Los Angeles, Los Angeles, California, United States of America; 8 Department of Pathology, University of California, San Francisco, San Francisco, California, United States of America; University of Cambridge, UNITED KINGDOM

## Abstract

**Background:**

Alzheimer disease (AD) is a progressive disorder that affects cognitive function. There is increasing support for the role of neuroinflammation and aberrant immune regulation in the pathophysiology of AD. The immunoregulatory human leukocyte antigen (HLA) complex has been linked to susceptibility for a number of neurodegenerative diseases, including AD; however, studies to date have failed to consistently identify a risk HLA haplotype for AD. Contributing to this difficulty are the complex genetic organization of the HLA region, differences in sequencing and allelic imputation methods, and diversity across ethnic populations.

**Methods and findings:**

Building on prior work linking the HLA to AD, we used a robust imputation method on two separate case–control cohorts to examine the relationship between HLA haplotypes and AD risk in 309 individuals (191 AD, 118 cognitively normal [CN] controls) from the San Francisco-based University of California, San Francisco (UCSF) Memory and Aging Center (collected between 1999–2015) and 11,381 individuals (5,728 AD, 5,653 CN controls) from the Alzheimer’s Disease Genetics Consortium (ADGC), a National Institute on Aging (NIA)-funded national data repository (reflecting samples collected between 1984–2012). We also examined cerebrospinal fluid (CSF) biomarker measures for patients seen between 2005–2007 and longitudinal cognitive data from the Alzheimer’s Disease Neuroimaging Initiative (*n* = 346, mean follow-up 3.15 ± 2.04 y in AD individuals) to assess the clinical relevance of identified risk haplotypes. The strongest association with AD risk occurred with major histocompatibility complex (MHC) haplotype A*03:01~B*07:02~DRB1*15:01~DQA1*01:02~DQB1*06:*02* (*p* = 9.6 x 10^−4^, odds ratio [OR] [95% confidence interval] = 1.21 [1.08–1.37]) in the combined UCSF + ADGC cohort. Secondary analysis suggested that this effect may be driven primarily by individuals who are negative for the established AD genetic risk factor, apolipoprotein E *(APOE*) ɛ4. Separate analyses of class I and II haplotypes further supported the role of class I haplotype A*03:01~B*07:*02* (*p* = 0.03, OR = 1.11 [1.01–1.23]) and class II haplotype DRB1*15:01- DQA1*01:02- DQB1*06:*02* (*DR15*) (*p* = 0.03, OR = 1.08 [1.01–1.15]) as risk factors for AD. We followed up these findings in the clinical dataset representing the spectrum of cognitively normal controls, individuals with mild cognitive impairment, and individuals with AD to assess their relevance to disease. Carrying A*03:01~B*07:*02* was associated with higher CSF amyloid levels (*p* = 0.03, β ± standard error = 47.19 ± 21.78). We also found a dose-dependent association between the *DR15* haplotype and greater rates of cognitive decline (greater impairment on the 11-item Alzheimer’s Disease Assessment Scale cognitive subscale [ADAS11] over time [*p* = 0.03, β ± standard error = 0.7 ± 0.3]; worse forgetting score on the Rey Auditory Verbal Learning Test (RAVLT) over time [*p* = 0.02, β ± standard error = −0.2 ± 0.06]). In a subset of the same cohort, dose of *DR15* was also associated with higher baseline levels of chemokine CC-4, a biomarker of inflammation (*p* = 0.005, β ± standard error = 0.08 ± 0.03). The main study limitations are that the results represent only individuals of European-ancestry and clinically diagnosed individuals, and that our study used imputed genotypes for a subset of HLA genes.

**Conclusions:**

We provide evidence that variation in the HLA locus—including risk haplotype *DR15*—contributes to AD risk. *DR15* has also been associated with multiple sclerosis, and its component alleles have been implicated in Parkinson disease and narcolepsy. Our findings thus raise the possibility that *DR15*-associated mechanisms may contribute to pan-neuronal disease vulnerability.

## Abstract 2

In a case–control study, Jennifer Yokoyama and colleagues present fine-mapping of the human leukocyte antigen genetic region to identify haplotypes associated with Alzheimer disease.

## Author summary

**Why was this study done?:**

- The human leukocyte antigen (HLA) region of the human genome encodes proteins that play an important role for the function of the immune system.
- Previous large genetic association studies have shown strong links between genes in this region and risk for Alzheimer disease.
- Although it is clear that the HLA region is linked to Alzheimer disease risk, the specific genes responsible for this have been difficult to determine because of the architecture of this complex genomic region.
- In addition, the patterns of variability in these genes differ widely in diverse ethnic and geographic populations, further complicating gene-mapping efforts.

**What did the researchers do and find?:**

- We used a large cohort of 11,690 white individuals to compare variants in HLA genes individually and in combination (termed haplotypes) between people who had Alzheimer disease and cognitively normal older adult controls.
- We found that several haplotypes were significantly associated with Alzheimer disease, including the haplotype A*03:01~B*07:02~DRB1*15:01*DQA1*01:02~DQB1*06:*02*.
- The components of this haplotype remained significant when separated into class I (A~B) and class II (DRB1~DQA1~DQB1) haplotypes and were associated with several clinical measures, including increased levels of a pathogenic protein in cerebrospinal fluid and decreased performance on two cognitive tests over time.

**What do these findings mean?:**

- Our results help identify specific variation in genes within the HLA region that may contribute to increased risk of Alzheimer disease.
- The class II haplotype that was associated with greater risk for Alzheimer disease (DRB1*15:01~DQA1*01:02~DQB1*06:*02*) in our cohort is also a significant risk factor for multiple sclerosis, suggesting a potential immune-mediated link between Alzheimer disease and other neurodegenerative diseases.
- Our results must be considered within the limitations that we only analyzed individuals of European ancestry, we included individuals who were only diagnosed with Alzheimer disease on a clinical basis and not confirmed upon autopsy, and we used imputed genetic information as opposed to direct sequencing data (which were unavailable).

## Introduction

Alzheimer disease (AD) is a progressive neurodegenerative disorder and has a global burden of approximately 46 million people worldwide, with prevalence projected to double over the next 20 y [1]. The hallmark features of the disease include the accumulation of amyloid plaques, tau neurofibrillary tangles, and neuronal destruction, leading to brain atrophy and loss of cognitive function. The etiology of these processes stems from synergistic interactions of environmental and genetic factors, many of which remain obscure and therefore complicate research efforts aimed at identifying efficacious therapies.

The three largest genetic contributors identified thus far are rare variants in amyloid precursor protein (*APP*) and presenilin 1 and 2 (*PSEN1*, *PSEN2*) [2]. These variants are uncommon, cause an early onset form of the disease, and typically segregate in an autosomal dominant fashion. Studies of late onset AD (typically defined as onset age >65 y) have demonstrated the risk of sequence variants such as the common ε4 allele of apolipoprotein E (*APOE*), rare variation in *TREM2* [3–7], and *MAPT* [8], as well as numerous common variants contributing modest AD risk [9], including single nucleotide polymorphisms (SNPs) in the following loci: *CR1*, *BIN1*, *INPP5D*, *MEF2C*, *CD2AP*, *ZCWPW1*, *NME8*, *EPHA1*, *CLU*, *PICALM*, *MS4A4*, *CELF1*, *FERMT2*, *ABCA7*, *CD33*, *CASS4*, *PTK2B*, *SORL1*, *SLC24A4-RIN3*, *DSG2*, and *HLA-DRB5/HLA-DRB1* [9,10]. However, there remain additional unexplained genetic contributions to nonfamilial forms of AD, suggesting polygenic contributors as well as the potential for epistatic and epigenetic interactions [11].

There is increasing support for the role of neuroinflammation in the etiology of AD as well as evidence that inflammatory processes are an early event in the brains of patients with AD [12]. Several studies have provided biochemical and histological evidence of classic immune components, including active microglia [13–15], complement factors [16,17], inflammatory cytokines [18], and C-reactive protein [19] within the parenchyma of AD brains. This is further supported by work in mouse models providing strong evidence for the role of complement-dependent destruction of synapses by phagocytic microglia prior to plaque deposition; similar mechanisms may even contribute to age-related cognitive decline [20,21]. Given these findings, there is a great deal of interest in identifying genetic determinants of inflammation related to AD susceptibility.

Located on chromosome 6p21, the major histocompatibility complex (MHC) is a dense region of approximately 150 genes that encode the human leukocyte antigen (HLA) immunoregulatory proteins [22]. Because of their proximity to each other, many of the MHC genes exist in linkage disequilibrium (LD) and are inherited as haplotypes with varying frequencies in global populations. MHC genes encode cell surface receptors and are classified based on their ability to present endogenous or exogenous antigens to T cells. MHC class I proteins exist on the surface of all nucleated cells and present fragments of antigens generated intracellularly to CD8+ T cells to induce a cytokine-mediated immune response. MHC class II molecules are only expressed by professional antigen-presenting cells, including B cells, macrophages, and microglia, and present exogenous material taken into the cell via endocytic vesicles to CD4+ T cells. Together, the diverse repertoire of the human immune system partly stems from the extremely polymorphic nature of the MHC class I and II regions.

Many associations are established between neurodegenerative and autoimmune diseases, specific class I and II alleles, and combinations of alleles (haplotypes) in the HLA region. Previous genome-wide association studies (GWASs), pleiotropic analyses, and meta-analyses by our group and others have investigated MHC susceptibility loci in a wide range of diseases, including AD [9,23,24]. However, because of the complex genetic organization of the HLA region and differences in the haplotype substructure of different ethnic populations, as well as differences in sequencing and allelic imputation methods, studies have yet to definitively elucidate which genes and specific alleles contribute to the observed association signals.

As mentioned, *HLA-DRB5/HLA-DRB1* has been implicated in numerous GWASs as a significant contributor to AD risk [9]. This prior work has established a significant association of the HLA locus to AD risk in over 75,000 individuals, yet the specific allele or alleles contributing to this association remain elusive. We thus used a robust HLA imputation method and case–control approach to fine-map the contributions of HLA polymorphisms and haplotypes to AD in over 11,500 patients and controls from independent cohorts from the University of California, San Francisco (UCSF) Memory and Aging Center (MAC) and the Alzheimer’s Disease Genetics Consortium (ADGC) (S1 File). We also examined longitudinal neuropsychological measures of cognitive function and cross-sectional biomarker data from cerebrospinal fluid (CSF) from the Alzheimer’s Disease Neuroimaging Initiative (ADNI) to assess the clinical relevance of identified risk haplotypes.

## Materials and methods

Participants were consented (as described below) for research in accordance with the Institutional Review Board at the University of California, San Francisco, and institutional review boards at each site for multicenter study data approved all aspects of this study as they fall under the purview of the respective research groups (ADNI and ADGC).

### Participants

#### UCSF MAC cohort

The participants included in this study were 309 white individuals over the age of 50 y, including 191 controls and 118 individuals with AD seen at the UCSF MAC between 1999–2012 who were genotyped as part of their participation in longitudinal research on neurodegenerative disease and healthy cognitive aging. DNA from the UCSF MAC cohort was collected from 2000–2012, and genotyping was performed in 2012. Because individuals are followed up longitudinally, we verified clinical diagnosis at the beginning of this study (May 2015). A multidisciplinary team of neurologists, neuropsychologists, and nurses performed a detailed evaluation on individuals with AD and established a diagnosis according to consensus criteria for AD [25]. Individuals included as controls underwent a similar assessment and were diagnosed as having normal cognition for their age. Participants who carried a known genetic risk variant in *APP*, *PSEN1*, or *PSEN2* were excluded from this study. Participants or surrogates completed written informed consent for all genetic research related to neurodegenerative disease and healthy cognitive aging during their initial visit in accordance with the Institutional Review Board at the University of California, San Francisco.

#### ADGC

The ADGC is an NIH-funded collection of GWAS data created for the goal of identifying genetic contributions to late-onset AD. Participants included in this study were from 30 merged datasets combined by Boehme, Mukherjee, Crane, and Kauwe and included 28,730 individuals carrying either an AD or cognitively normal control clinical diagnosis [26] A list of the datasets and basic information is included in S1 Table; full details on the datasets and the merging process are available at http://kauwelab.byu.edu/Portals/22/adgc_combined_1000G_12032014.pdf [26]. Analyses were limited to white individuals for maximum statistical power to reduce potential for confounding due to the known population-based contribution to diversity in the HLA region. Participants were recruited and seen between 1984–2012. Written informed consent for genetic studies falling under the purview of the ADGC was obtained from all study participants, and institutional review boards at each site approved all aspects of this study. Specific consent for this study was obtained from the ADGC based on an application describing the proposed work.

#### ADNI

We also utilized data from 346 individuals recruited for participation in the ADNI study with data from SNP genotyping and longitudinal cognitive scores. All individuals included in this study had a minimum of two clinic assessments. At baseline, 120 individuals were cognitively normal (CN) older adults, 113 individuals were diagnosed with mild cognitive impairment (MCI), and 113 with AD. Of these, 163 individuals also had CSF measurements of plasma biomarkers available (S2 Table). The ADNI cohort is well characterized and has been used in previously published studies [27–29]. The clinical severity of symptoms in the MCI and AD groupings was measured using the Clinical Dementia Rating sum of boxes (CDR-SB) [30]. A clinician diagnosed each participant using a structured protocol that utilized clinical judgment and neuropsychological tests that are provided in S1 Methods. The mean follow-up time was 3.15 ± 2.04 y for control participants (*n* = 91), 2.39 ± 1.71 y for participants with MCI (*n* = 148), and 1.37 ± 0.75 y for patients with AD (*n* = 69). Written informed consent was obtained from all study participants for research studies falling under the purview of ADNI, and the University of California, San Francisco Institutional Review Board approved all aspects of this study.

### Genotype acquisition

#### UCSF MAC cohort

Patient and control genotypes were obtained via genotyping on the Illumina Omni1-Quad array (Illumina, San Diego, California) using manufacturer’s instructions. *APOE* genotype was determined with a TaqMan Allelic Discrimination Assay for the two SNPs, rs429358 and rs7412, on an ABI 7900HT Fast Real-Time PCR system (Applied Biosystems, Foster City, California) using the manufacturer's instructions.

#### ADGC

Details of genotyping in the 30 datasets that comprise the combined ADGC dataset are available online [26] and partially described in previously published papers [10].

#### ADNI

Haplotypes were determined using genotypes from the Human610-Quad BeadChip (Illumina, San Diego, California) as previously described [31]. *APOE* genotypes were determined by Cogenics (now Beckman Coulter; Pasadena, California).

### CSF biomarker measurements

#### ADNI

Baseline CSF biomarkers levels were measured using the Human DiscoveryMAP panel developed by Rules Based Medicine (Myriad RBM; Austin, Texas). The Human DiscoveryMAP panel is commercially available and measures a collection of metabolic, lipid, inflammatory, and other AD-relevant indicators. We limited our analyses to 28 immune proteins in the panel that were associated with inflammatory or immune processes (S1 List). The samples were processed and analyzed by Myriad RBM and checked for quality by the ADNI Biomarker core. CSF amyloid β 1–42 was measured using the AlzBio3 Luminex xMAP immunoassay (Innogenetics, Ghent, Belgium) according to previously described methods [32]. This method utilizes a monoclonal antibody specific for amyloid β 1–42 that is chemically bonded to color-coded beads along with analyte-specific detector antibodies. Additional details are available in S1 Methods.

### Clinical assessments

#### ADNI

In this study, we analyzed two neuropsychological measures of cognitive function and one measure of clinical severity in ADNI participants. The Rey Auditory Verbal Learning Test (RAVLT) [33] is a test of verbal memory. It begins with the administrator reading a list of 15 unrelated words to the participant, who is then asked to verbally repeat as many of the words as they can. This happens for a total of five learning trials, and the administrator records the number of words correctly recalled after each trial. The test administrator then reads a set of 15 new words to the participant (interference word list), and, immediately following this, the participant is asked to recall as many of the first list of words as possible (immediate recall score). After a 30-min delay during which unrelated tests are administered, the participant is asked to recall as many words as possible from the initial list (delayed recall score). The RAVLT “forgetting score” is calculated as the difference between immediate recall versus delayed recall scores [33]. The forgetting score remains relatively stable over time in individuals with consistent memory function; the forgetting score tends to get smaller as the number of recalled items decreases. The 11-item Alzheimer’s Disease Assessment Scale (ADAS) cognitive subscale assesses learning and memory, orientation, and several aspects of language including production, comprehension, and constructional and ideational praxis [34,35]. Higher scores indicate more impairment. Finally, the Clinical Dementia Rating (CDR) scale is a measure of three cognitive domains (memory, problem solving, and orientation) and three functional domains (self-care, community engagement, and hobbies). Information is collected directly from the study participant, as well as from a study informant. The scores for the six domains are combined into the CDR sum of boxes (CDR-SB) score [36].

### Statistical analysis

#### Cohort demographic summary statistics

Summary statistics for participants’ age, sex, age of onset, and *APOE* ε4 carrier status were calculated using R.

#### Imputation of HLA alleles

HLA genotypes were derived from chromosome 6 SNP data using an imputation program, HLA Genotype Imputation with Attribute Bagging (HIBAG) v1.3, which calculates predictions of genotype by averaging HLA-type posterior probabilities over an ensemble of classifiers built on bootstrap samples [37]. It relies on a training set of known HLA and SNP genotypes. We imputed the following HLA genes: *A*, *B*, *DRB1*, *DQA1*, and *DQB1*. For the UCSF MAC cohort and the ADGC merged dataset, a training set for four-digit resolution using ethnic-specific models for Europeans based on Omni1_Quad_v1_0_H was used. For the ADNI cohort clinical biomarker analyses, we used four-digit resolution ethnic-specific models for Europeans derived from Illumina Human610-Quad v1.0.

#### Quality control of HLA imputation

Based on the distribution of posterior probabilities for each of the five imputed alleles (see S1 Fig), we chose a call threshold (CT) of 0.75. As previous studies have shown that a CT of 0.5 leads to HIBAG prediction accuracies of 94.8%–99.2% for individuals of European ancestry [38], we expect our more stringent CT will correspond to similar or higher HIBAG prediction accuracies based on assumed accuracy of imputed ADGC SNPs. After excluding samples with any imputation probability below this cutoff at any locus, our final ADGC cohort size was 11,381.

#### Calculating locus and haplotype odds ratios (ORs)

OR estimates for patients with AD and cognitively normal controls were calculated using a statistical package designed to specifically probe associations with the HLA (BIGDAWG), including tests of Hardy-Weinberg equilibrium and case–control association analyses for haplotypes as previously described [39]. Analyses were performed for each cohort (UCSF + ADGC) separately and in combination. As this was a fine-mapping study based on a previous genome-wide significant, and replicated, finding at *HLA-DRB5;* and considering that this study represents a first analysis of the highly polymorphic HLA region in the context of AD, a complex disease, we did not require a multiple testing correction. To strike a balance between reducing Type I error while also allowing for full exploration of the loci underlying this association with the MHC region, we implemented a stepwise assessment of HLA gene contributions to AD: using allelic information, we established a priori significance at *p* < 0.05 at the haplotype level based on the prior GWAS-significant results. We then examined the contingency table from which the haplotype result was derived to identify the specific allele(s) contributing to the association signal. We accepted allele-level significance at *p* < 0.05 given the haplotype-level significance [40,41]. Based on a sample size of 11,690 in our combined UCSF+ADGC cohort, with 326 degrees of freedom and an alpha of 0.05, we had 64.1% power to detect an OR of 1.21 based on the haplotype frequencies of AD versus cognitively normal controls for the top associated five-allele haplotype.

#### Biomarker and cognitive data

Discrete and continuous demographic variables were compared across the ADNI cohort using chi-squared and ANOVA analyses, respectively. Linear mixed effects models were used to assess the relationship between the risk haplotype of interest and changes in the longitudinal cognitive measurements, ADAS and RAVLT, while controlling for baseline and time interactions of age, sex, education, baseline CDR-SB score (to account for baseline differences in clinical severity/diagnosis), and *APOE* ε4 carrier status. Use of linear mixed effects models allowed us to account for variable data missingness across participants by estimating subject-specific slopes. This enabled us to estimate cognitive changes for each individual despite varying numbers of visits. Missing data were omitted from the analyses, and all participants were required to have at least two time points to be included in the analysis. All interactions and main effects were modeled as fixed effects with random slopes and intercepts across individuals. The main effects of all variables were included in all longitudinal analyses but have been omitted from the definitions below to improve their clarity.

The linear mixed effects model for ADAS11 scores was defined as follows:

$$\Delta \;ADAS=\beta_{0}+\beta_{1}\Delta t+\beta_{2}\text{DR}15*\Delta t+\beta_{3}\text{Age}*\Delta t+\beta_{4}\text{Sex}*\Delta t+\beta_{5}\text{Education}*\Delta t+\beta_{6}\text{CDR-SB}*\Delta t+\beta_{7}APOE\;\varepsilon 4*\Delta t+e.$$

The linear mixed effects model for the RAVLT forgetting score was defined as follows:

$$\Delta \;RAVLT=\beta_{0}+\beta_{1}\Delta t+\beta_{2}\text{DR}15*\Delta t+\beta_{3}\text{Age}*\Delta t+\beta_{4}\text{Sex}*\Delta t+\beta_{5}\text{Education}*\Delta t+\beta_{6}\text{CDR-SB}*\Delta t+\beta_{7}APOE\;\varepsilon 4*\Delta t+e.$$

#### Cross-sectional CSF biomarker analyses

Linear models were used to test for an association between baseline CSF biomarker levels and the haplotype of interest. We controlled for age, sex, education, baseline CDR-SB score (to account for baseline differences in clinical severity/diagnosis), and *APOE* ε4 dosage.

## Results

### Five-allele haplotype analysis implicated *DR15* in AD risk

The discovery UCSF cohort consisted of 309 individuals with clinically diagnosed AD and cognitively normal older adult controls (Table 1). Because of the small sample size, all imputed alleles were included in the haplotype analysis (HLA *A*, *B*, *DRB1*, *DQA1*, and *DQB1*). We performed association analysis on the four haplotypes with sufficient frequency in this small cohort. Of these four, one showed a significant association with AD risk: HLA A*02:01~B*07:02~DRB1*15:01~DQA1*01:02~DQB1*06:*02* (OR = 3.69; 95% confidence interval [CI] 1.16–13.69; *p* = 0.01) (Table 2).

**Table 1:** Cohort demographics.

Cohort	*n*	CN/AD	% Male	Age at onset
UCSF	309	191/118	46.3%	72.7 ± 9.0
ADGC	11,381	5,728/5,653	41.4%	74.0 ± 7.7

Mean ± standard deviation of age of onset indicates age of first reported symptoms. ADGC, Alzheimer’s Disease Genetic Consortium merged dataset; UCSF, University of California, San Francisco Memory and Aging Center.

**Table 2:** Five-allele haplotype risk associations in two clinical cohorts and combined dataset.

Cohort Information	Haplotype: *A~B~DRB1~DQA1~DQB1*	OR (95% CI)	*p-*Value	Frequency CN	Frequency AD
**UCSF** *n* = 309 191 CN; 118 AD 4 haplotypes analyzed	*01*:*01~08*:*01~03*:*01~05*:*01~02*:*01*	*0*.*87 (0*.*45–1*.*64)*	*0*.*66*	*0*.*0864*	*0*.*0763*
	**02:01~07:02~15:01~01:02~06:02**	**3.69 (1.16–13.69)**	**0.01**	**0.0131**	**0.0466**
	*02*:*01~08*:*01~03*:*01~05*:*01~02*:*01*	*0*.*64 (0*.*15–2*.*26)*	*0*.*45*	*0*.*0262*	*0*.*0170*
	*03*:*01~07*:*02~15*:*01~01*:*02~06*:*02*	*0*.*72 (0*.*28–1*.*68)*	*0*.*42*	*0*.*0524*	*0*.*0381*
**ADGC** *n* = 11,381 5,728 CN; 5,653 AD 318 haplotypes analyzed	01:01~08:01~07:01~02:01~03:03	0.43 (0.16–1.02)	0.04	0.0017	0.0007
	*02*:*01~07*:*02~15*:*01~01*:*02~06*:*02*	*1*.*08 (0*.*93–1*.*25)*	*0*.*30*	*0*.*0320*	*0*.*0345*
	**02:01~13:02~07:01~02:01~02:02**	**0.66 (0.50–0.89)**	**4.2 x 10**^**-3**^	**0.0107**	**0.0072**
	02:01~15:01~07:01~02:01~02:02	0.39 (0.14–0.99)	0.03	0.0016	0.0006
	02:01~44:02~13:01~01:03~06:03	1.44 (1.03–2.03)	0.03	0.0054	0.0078
	02:01~57:01~07:01~02:01~03:03	1.31 (1.01–1.69)	0.04	0.0095	0.0124
	03:01~07:02~12:01~05:05~03:01	0.30 (0.09–0.84)	0.01	0.0015	0.0004
	**03:01~07:02~15:01~01:02~06:02**	**1.22 (1.08–1.38)**	**8.5 x 10**^**-4**^	**0.0472**	**0.0570**
	11:01~35:01~07:01~02:01~02:02	0.31 (0.07–1.01)	0.03	0.0011	0.0004
	**24:02~38:01~13:01~01:03~06:03**	**0.14 (0.02–0.63)**	**2.9 x 10**^**-3**^	**0.0012**	**0.0002**
	24:02~44:05~01:01~01:01~05:01	4.56 (0.94–43.38)	0.03	0.0002	0.0008
	29:02~58:01~08:04~04:01~04:02	4.56 (0.94–43.38)	0.03	0.0002	0.0008
	68:01~44:02~01:01~01:01~05:01	1.96 (0.99–4.04)	0.04	0.0012	0.0024
**ADGC + UCSF** *n* = 11,690 5,919 CN; 5,717 AD 326 haplotypes analyzed	01:01~08:01~07:01~02:01~03:03	0.43 (0.16–1.03)	0.04	0.0016	0.0007
	02:01~07:02~01:01~01:01~05:01	1.78 (0.98–3.32)	0.04	0.0016	0.0029
	*02*:*01~07*:*02~15*:*01~01*:*02~06*:*02*	*1*.*08 (0*.*94–1*.*25)*	*0*.*28*	*0*.*0317*	*0*.*0342*
	**02:01~13:02~07:01~02:01~02:02**	**0.66 (0.49–0.88)**	**3.8 x 10**^**-3**^	**0.0106**	**0.0070**
	02:01~15:01~07:01~02:01~02:02	0.38 (0.13–0.94)	0.02	0.0016	0.0006
	02:01~18:01~07:01~02:01~02:02	3.42 (0.88–19.35)	0.047	0.0003	0.0009
	02:01~44:02~13:01~01:03~06:03	1.49 (1.07–2.10)	0.01	0.0052	0.0078
	02:01~57:01~07:01~02:01~03:03	1.32 (1.02–1.72)	0.03	0.0092	0.0121
	03:01~07:02~12:01~05:05~03:01	0.32 (0.09–0.92)	0.02	0.0014	0.0004
	**03:01~07:02~15:01~01:02~06:02**	**1.21 (1.08–1.37)**	**9.6 x 10**^**-4**^	**0.0476**	**0.0573**
	11:01~35:01~07:01~02:01~02:02	0.29 (0.07–0.93)	0.02	0.0012	0.0003
	**24:02~38:01~13:01~01:03~06:03**	**0.15 (0.02–0.64)**	**3.2 x 10**^**-3**^	**0.0012**	**0.0002**
	24:02~44:05~01:01~01:01~05:01	5.13 (1.09–48.17)	0.02	0.0002	0.0009
	29:02~58:01~08:04~04:01~04:02	4.62 (0.96–43.93)	0.03	0.0002	0.0008
	68:01~44:02~01:01~01:01~05:01	1.91 (0.96–3.95)	0.048	0.0012	0.0023

All analyzed haplotype association results (regardless of significance) are reported for the UCSF cohort, with the significant (*p* < 0.05) finding in bold. Nonsignificant results (*p* > 0.05) are shown in italics. For the ADGC cohort alone and the combined UCSF + ADGC analysis, all significant (*p* < 0.05) haplotype association results are reported, in addition to the results for the single significant haplotype from the UCSF cohort (not significant in the ADGC analysis [*p* = 0.30] or in the ADGC+UCSF analysis [*p* = 0.28] in italics). The top three most significant ADGC and ADGC+UCSF findings are highlighted with a light blue background. In addition to odds ratio (OR) with 95% confidence interval (CI), a breakdown of the haplotype frequency in individuals with Alzheimer disease (AD) versus cognitively normal (CN) older adult controls is also provided.

After quality control, 11,381 individuals were available for analysis in the validation ADGC cohort (Table 1). Of the 318 haplotypes available for analysis, 12 five-allele haplotypes were significantly associated with AD risk (*p* < 0.05, Table 2). The strongest association was HLA A*03:01~B*07:02~DRB1*15:01~DQA1*01:02~DQB1*06:*02* (OR = 1.22 [1.08–1.38], *p* = 8.5 x 10^−4^). This haplotype differed from the UCSF finding by one allele, at *HLA-A*. The third most significant haplotype association in the ADGC cohort was A*02:01~B*13:02~DRB1*07:01~DQA1*02:01~DQB1*02:*02*, which showed a protective effect, (OR = 0.66 [0.50–0.89], *p* = 4.2 x 10^−3^). This haplotype shared the *HLA-A* allele associated with AD risk in the UCSF discovery analysis. The full A*02:01~B*07:02~DRB1*15:01~DQA1*01:02~DQB1*06:*02* haplotype associated with AD in the UCSF cohort was not significant in the ADGC cohort (*p* = 0.30).

In combined analysis of both the UCSF and ADGC cohorts, 326 haplotypes were available for analysis (additional haplotypes beyond the 4 + 318 haplotypes analyzed in the separate UCSF and ADGC cohorts resulted when sufficient numbers of AD and CN controls for rare haplotypes became available in the combined UCSF + ADGC dataset). HLA A*03:01~B*07:02~DRB1*15:01~DQA1*01:02~DQB1*06:*02* (OR = 1.21 [1.08–1.37), *p* = 9.6 x 10^−4^) and A*02:01~B*13:02~DRB1*07:01~DQA1*02:01~DQB1*02:*02*, [OR = 0.66 [0.49–0.88], *p* = 3.8 x 10^−3^] remained as two of the three most significant associations with AD (Table 2). Locus-level analyses of the combined cohort showed independent AD associations of B*07:*02*, DRB1*15:*01*, DQA1*01:*02*, and DQB1*06:*02* (Table 3).

**Table 3:** Individual alleles with significant risk associations in combined cohort.

**Class I Loci**	**OR (95% CI)**	***p-*Value**	**Frequency CN**	**Frequency AD**	**European population frequency estimate**
A*23:01	0.81 (0.67–0.98)	0.03	0.0223	0.0182	0.0168
A*33:03	1.97 (0.97–4.21)	0.04	0.0011	0.0022	0.0013
B*07:02	**1.07 (1.00–1.15)**	**0.04**	**0.1629**	**0.1727**	**0.1400**
B*15:01	0.87 (0.78–0.98)	0.02	0.0627	0.0553	0.0665
B*41:01	2.15 (1.00–4.88)	0.03	0.0009	0.0020	0.0038
B*57:01	1.15 (1.01–1.30)	0.03	0.0406	0.0462	0.0383
**Class II Loci**	**OR (95% CI)**	***p-*Value**	**Frequency CN**	**Frequency AD**	**European population frequency estimate**
DRB1*15:01	**1.08 (1.01–1.15)**	**0.03**	**0.1795**	**0.1907**	**0.1444**
DQA1*01:02	**1.06 (1.00–1.13)**	**0.04**	**0.2373**	**0.2487**	**not available**
DQB1*06:02	**1.08 (1.01–1.15)**	**0.03**	**0.1782**	**0.1895**	**0.1425**

All significant loci results (*p* < 0.05) for combined UCSF and ADGC cohort (*n* = 11,690). Alleles present in one of the top three most significant (*p* < 0.01) five-allele haplotypes (Table 2) are shown in bold. The number of analyzed alleles differed by loci (A: *n* = 23, B: *n* = 39, DQB1: *n* = 27, DQA1: *n* = 13, DQB1: *n* = 15). In addition to OR with 95% CI, a breakdown of allele frequency in individuals with Alzheimer disease (AD) versus cognitively normal (CN) older adult controls is also provided in addition to the expected frequency in populations of European descent [42]

### Five-allele haplotype contributed to AD risk independently of APOE ɛ4 and may be driven by ɛ4-negative individuals

We next assessed whether the strong genetic AD risk factor *APOE* ɛ4 can account for the most significant five-allele haplotype association we identified in the combined UCSF+ADGC cohort. We recoded individuals as carriers or noncarriers of the A*03:01~B*07:02~DRB1*15:01~DQA1*01:02~DQB1*06:*02* haplotype and assessed in a logistic regression framework the independent contributions of the risk haplotype, *APOE* ɛ4 carrier status, and whether there was an interaction between the two. As expected, *APOE* ɛ4 was strongly associated with AD risk (*p* < 2 x 10^−16^). The five-allele risk haplotype remained a significant contributor to AD risk (*p* = 0.036), but there was no statistically significant interaction between haplotype and *APOE* ɛ4 (*p* = 0.19). However, dividing the cohort by *APOE* ɛ4 carrier status showed that the frequency of the A*03:01~B*07:02~DRB1*15:01~DQA1*01:02~DQB1*06:*02* haplotype was higher only in individuals with AD who are negative for ɛ4 (Table 4). Analysis of variance performed separately in ɛ4 carriers and noncarriers resulted in a significant association of the five-allele haplotype with AD only in ɛ4-negative individuals (*p* = 0.036 in ɛ4 noncarriers; *p* = 0.90 in ɛ4 carriers).

**Table 4:** AD/CN distribution by *APOE* ɛ4 and A*03:01~B*07:02~DRB1*15:01~DQA1*01:02~DQB1*06:*02* haplotype carrier status.

Haplotype status	*APOE* ɛ4+ AD	*APOE* ɛ4+ CN	*APOE* ɛ4- AD	*APOE* ɛ4- CN
A*03:01~B*07:02~DRB1*15:01~DQA1*01:02~DQB1*06:*02* carriers	0.726	0.274	0.391	0.609
A*03:01~B*07:02~DRB1*15:01~DQA1*01:02~DQB1*06:*02* noncarriers	0.723	0.277	0.344	0.656
*p*-Value (ANOVA)	0.901		**0.036**	

Alzheimer disease (AD)/cognitively normal (CN) distribution by *APOE* ɛ4 status and risk haplotype carrier status. Of the full cohort, 9,517 individuals had information on *APOE* genotype and were included in this analysis. Individuals with either one or two ɛ4 alleles were classified as *APOE* ɛ4 positive.

### Separate class I and class II haplotype analyses corroborated A*03:01~B*07:*02* and *DR15* in AD risk

Given the different roles of HLA receptors in recognizing endogenous (class I) or exogenous (class II) ligands, we also assessed class I (*HLA A~B*) and class II (*HLA DRB1~DQA1~DQB1*) haplotypes separately for their role in AD risk in the combined UCSF+ADGC cohort. Of 202 analyzed class I haplotypes, ten two-allele haplotypes were significantly associated with AD (*p* < 0.05), including A*03:01~B*07:*02* (*p* = 0.03, OR = 1.1 [1.0–1.2]) (Table 5). Only one three-allele class II haplotype (out of 30 analyzed) was associated with AD risk, *DR15* (*p* = 0.025, OR = 1.1 [1.0–1.2]). Together, these two separate haplotypes represent the most strongly associated five-allele haplotype identified in the combined analysis.

**Table 5:** Separate class I and class II haplotypes with significant risk associations in combined cohort.

**Class I Haplotypes *A~B***	**OR (95% CI)**	***p-*Value**	**Frequency CN** **Controls**	**Frequency AD**
01:01~57:01	1.21 (1.01–1.46)	0.04	0.0187	0.0225
**02:01~13:02**	**0.66 (0.51–0.86)**	**1.4 x 10**^**−3**^	**0.0130**	**0.0087**
**03:01~07:02**	**1.11 (1.01–1.23)**	**0.03**	**0.0703**	**0.0777**
03:01~15:01	0.63 (0.45–0.87)	4.1 x 10^−3^	0.0083	0.0052
11:01~15:01	0.54 (0.29–0.99)	0.03	0.0029	0.0016
**24:02~38:01**	**0.36 (0.13–0.88)**	**0.01**	**0.0017**	**0.0006**
26:01~39:01	0.07 (0–0.48)	9.4 x 10^−4^	0.0012	0.0001
26:01~44:02	0.15 (0.02–0.64)	3.2 x 10^−3^	0.0012	0.0002
32:01~14:02	4.62 (0.96–43.93)	0.03	0.0002	0.0008
68:01~40:01	0.48 (0.30–0.75)	7.0 x 10^−4^	0.0054	0.0026
**Class II Haplotypes *DRB1~DQB1~DQA1***	**OR (95% CI)**	***p-*Value**	**Frequency CN**	**Frequency AD**
**15:01~01:02~06:02**	**1.08 (1.01–1.15)**	**0.03**	**0.1781**	**0.1894**

All significant (*p* < 0.05) class I (*A~B*) and class II (*DRB1~DQA1~DQB1*) haplotypes for combined UCSF and ADGC cohorts (*n* = 11,690). Class I and class II haplotypes present in one of the top three most significant (*p* < 0.01) five-allele haplotypes (Table 2) are shown in bold. In total, 202 class I haplotypes and 30 class II haplotypes were analyzed. In addition to OR with 95% CI, a breakdown of allele frequency in individuals with Alzheimer disease (AD) versus cognitively normal (CN) older adult controls is also provided.

### Class I haplotype A*03:01~B*07:*02* was associated with baseline CSF amyloid levels

We utilized a subset of the ADNI cohort with genetic and cognitive data available to assess the disease-specific relevance of the class I A*03:01~B*07:*02* and class II *DR15* haplotypes across the AD spectrum, including cognitively normal controls, individuals with MCI, and those with AD. We analyzed the two haplotypes separately to assess whether class I and class II risk-associated haplotypes were correlated with similar or different clinical measures of AD. The cohort was balanced with respect to age, sex, and haplotype distributions (Table 6). The cohort was significantly different with respect to education and number of time points and showed expected differences in CDR-SB baseline score, *APOE* ɛ4 carrier status, ADAS baseline score, and RAVLT forgetting baseline score.

**Table 6:** Summary statistics for ADNI participants with longitudinal cognitive measures.

	CN	MCI	AD	*p*-Value
*n*	120	113	113	
Age (years)	75.6 ± 4.87	74.0 ± 6.17	75.9 ± 6.72	NS
Sex (% female)	45.8%	35.4%	47.8%	NS
Education (years)	16.0 ± 2.7	15.5 ± 2.9	14.6 ± 3.0	<0.001
CDR-SB score	0.02 ± 0.1	1.7 ± 0.9	4.3 ± 1.7	<0.001
*APOE* ɛ4 carrier (%)	31.7%	67.3%	69.0%	<0.001
Haplotype dose (number of single / number of double)	33/0	30/3	33/3	NS
Time points	7.4 ± 2.8	6.6 ± 2.4	3.8 ± 0.8	<0.001
ADAS score (baseline)	6.0 ± 2.9	12.2 ± 4.1	18.5 ± 6.1	<0.001
RAVLT forgetting score (baseline)	3.5 ± 2.8	4.8 ± 2.3	4.5 ± 1.9	<0.001

Descriptive data are summarized by diagnostic category. Values represent the mean ± standard deviation and the percent or number of participants in a given diagnostic category. Two-tailed *p*-values were from analysis of variance (continuous traits) or chi-square (categorical values) tests by diagnostic group. AD, Alzheimer disease; ADAS, Alzheimer’s Disease Assessment Scale; ADNI, Alzheimer’s Disease Neuroimaging Initiative; CDR-SB, Clinical Dementia Rating sum of boxes; CN, cognitively normal; MCI, mild cognitive impairment; NS, not significant (*p* > 0.05); RAVLT, Rey Auditory Verbal Learning Test.

**Fig 1:**
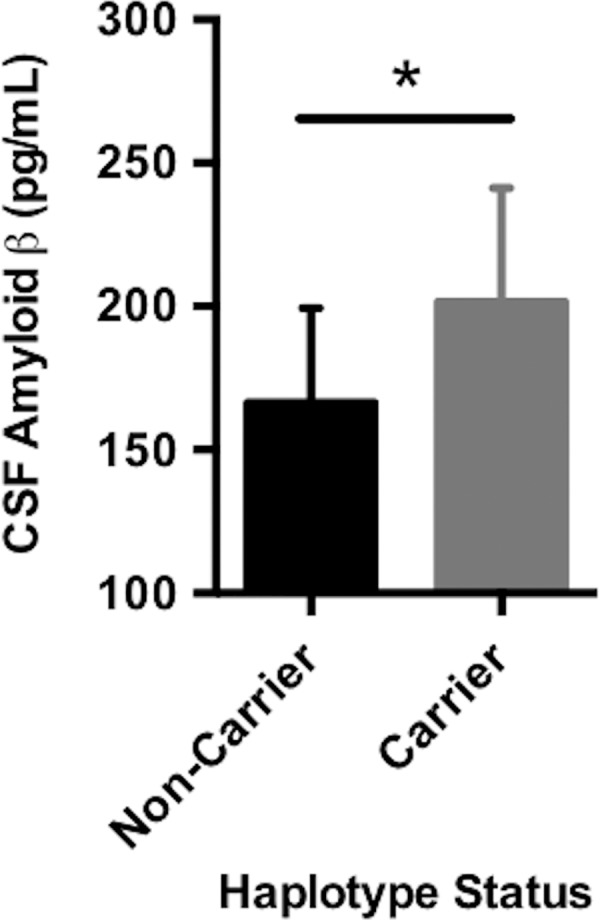
Carrying the A*03:01~B*07:*02* risk haplotype was associated with CSF (cerebrospinal fluid) amyloid β. CSF amyloid β levels were on average higher in carriers of the A*03:01~B*07:*02* haplotype, suggesting that haplotype carriers may have lower average intracranial amyloid pathological burden compared to noncarriers. The plotted points are best linear unbiased predictions from a multiple regression model, which controlled for age, sex, education, CDR-SB score, and *APOE* ɛ4 status. Data shown are the mean ± standard deviation (SD).

Carrying A*03:01~B*07:*02* was associated with higher baseline levels of amyloid β as measured in CSF (Fig 1, *p* = 0.01). Traditionally, CSF amyloid levels are inversely correlated with amyloid burden in the brain; our results suggest that carrying A*03:01~B*07:*02* is correlated with lower amyloid levels in the brain [43]. This is observed despite the fact that there were no statistically significant differences in baseline clinical or biomarker measures in patients with versus without the risk haplotype (S3 Table, S2 Fig). A*03:01~B*07:*02* was not associated with any other baseline measures and was not associated with change in longitudinal measures over time.

### *DR15* risk haplotype correlated with worse cognitive decline and greater baseline inflammation across the AD spectrum

**Fig 2:**
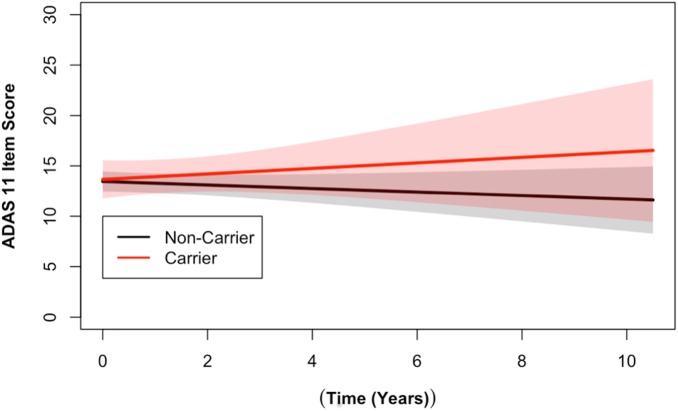
*DR15* haplotype carriers showed greater change over time on the ADAS cognitive assessment when compared to noncarriers. Longitudinal ADAS 11-item cognitive subscale scores from the ADNI cohort are shown. The ADAS broadly measures cognitive functions impaired in AD [34], with higher scores representing more cognitive impairment. *DR15* haplotype carriers (in red) showed worse cognitive function over time when compared to noncarriers (in black) (*p* = 0.03). The plotted data represent the best linear unbiased prediction results from the regression model specified (see Methods) with 95% CIs (shaded regions).

**Fig 3:**
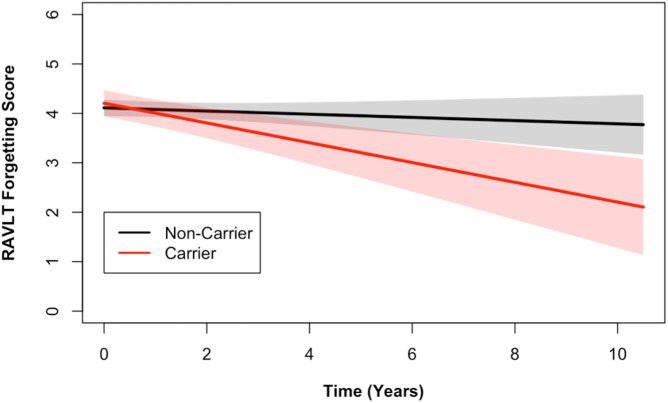
*DR15* haplotype carriers declined more on the RAVLT forgetting score when compared to noncarriers. Longitudinal RAVLT measurements from the ADNI cohort are shown. The RAVLT forgetting score is defined as the difference between the delayed recall and immediate recall scores on the RAVLT and represents a measure of memory consolidation. Over time, *DR15* risk haplotype carriers showed more change on the forgetting score (i.e., more forgetting) than noncarriers. The plotted data represent the best linear unbiased prediction results from the regression model specified (see Methods) with 95% CIs (shaded regions).

Longitudinal analysis of cognitive data identified a statistically significant association between the number of alleles of the *DR15* risk haplotype and ADAS cognitive scores (*p* = 0.03), as well as with RAVLT forgetting scores (*p* = 0.02, S4 Table). The *DR15* haplotype was associated with worse decline over time on both measures, corresponding to increasing longitudinal ADAS cognitive scores and decreasing longitudinal RAVLT forgetting scores over time (shown relative to noncarriers in Figs 2 and 3). *DR15*-associated changes in cognitive trajectory occurred despite the fact that there were no baseline differences in clinical severity or cognitive function in patients with AD based on *DR15* carrier status (S5 Table). In addition, baseline biomarker measures most relevant to AD were similar in both patients with AD who are *DR15* carriers and those who are noncarriers (S5 Table, S3 Fig), indicating that all patients had equivalent baseline disease severity.

**Fig 4:**
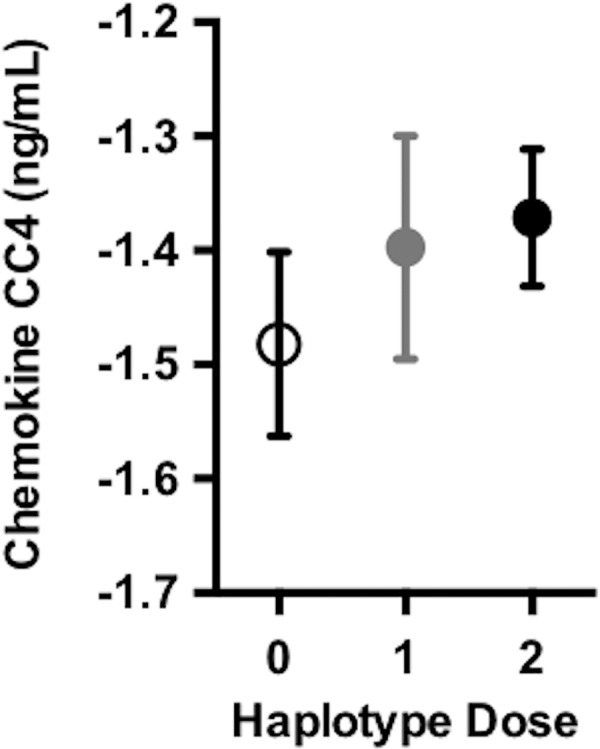
*DR15* dosage was associated with higher baseline levels of chemokine CC4. As the number of *DR15* risk haplotype alleles increases, there were higher average levels of chemokine CC4, suggesting higher levels of inflammation at baseline. Chemokine CC4 levels are quality controlled and transformed as described in S1 Methods. The plotted points are partial residuals with 95% confidence bands provided in shading.

In a subset of individuals who also had baseline CSF data available (S2 Table), we tested whether the *DR15* risk haplotype altered any biomarker measures of immunological function and inflammation. We tested 28 analyte levels related to immune function and inflammation (S1 List). At baseline, there was an association between chemokine CC-4 (CC4) and age (*p* = 0.02, S4 Fig), as well as CC4 with dose of *DR15* risk haplotype (*p* = 5.18 x 10^−3^, Fig 4, S6 Table). Although not reaching strict statistical significance after adjustment for the 28 biomarkers tested (at Bonferroni adjusted *p* < 1.79 x 10^−3^), this analysis provides suggestive biomarker evidence of heightened baseline inflammation in individuals carrying the *DR15* risk haplotype.

### HLA haplotype risk effects differed by sex

Given previous reports of greater risk effects of *DR15* in female patients with multiple sclerosis (MS) [44] and the stronger effect of *APOE* ɛ4 in females [45], we assessed whether men versus women showed similar or different HLA haplotype associations with AD risk. When split by sex, two of the three most significant five-allele haplotypes from the combined sex analysis were significant in an individual sex. The five-allele haplotype A*03:01~B*07:02~DRB1*15:01~DQA1*01:02~DQB1*06:*02* was significant only in men (OR 1.31 [1.09–1.58], *p* = 0.0035) (S7 Table). However, A*02:01~B*13:02~DRB1*07:01~DQA1*02:01~DQB1*02:*02* was significant only in women (OR 0.68 [0.46–0.99], *p* = 0.034) (S7 Table). Similar findings appeared in separate class I and class II haplotype analyses. Only men showed significant associations with class I haplotype A*03:01~B*07:*02* (*p* = 0.027), and only women showed significant associations with A*02:01~B*13:*02* (*p* = 0.0049) (S8 Table). Finally, class II haplotype *DR15* was only significantly associated with AD risk in men (*p* = 0.01) (S8 Table). Locus-level analyses were consistent, with only men showing significant associations with ten alleles, including B*07:*02* (*p* = 0.013), DRB1*15:*01* (*p* = 0.0096), DQA1*01:*02* (*p* = 0.029), and DQB1*06:*02* (*p* = 0.01) (S9 Table). There were four individual alleles associated with AD risk in women, none of which were components of any of the top three significant five-allele haplotypes in the combined sex analysis (S9 Table).

### Iterative subanalyses corroborate role of HLA-A*03:01~B*07:02~DRB1*15:01~DQA1*01:02~DQB1*06:*02* in AD

To attempt to alleviate concern over possible Type I error in this analysis, we randomly split the combined ADGC+UCSF cohort ten times (maintaining the same proportion of AD:controls) and reran the five-allele haplotype analysis in the 20 resulting (smaller) cohorts. Two of the top-associated five-allele haplotypes showed *p*-values < 0.05 in over half of the randomly split analyses (S10 Table), which was more than any of the other “top” haplotypes from the original analysis. This included the one we focused on in this study (A*03:01~B*07:02~DRB1*15:01~DQA1*01:02~DQB1*06:*02*), which showed significance in 11 iterations of the randomly split analysis, with *p*-values from 0.026–0.0001 and ORs of 1.21–1.40, further corroborating the contributions of this haplotype and its subcomponents to AD risk.

## Discussion

In a total of over 11,000 individuals, we found evidence suggesting that the five-allele HLA haplotype A*03:01~B*07:02~DRB1*15:01~DQA1*01:02~DQB1*06:*02* is a risk factor for AD and that this effect may be driven by men who do not carry the major AD risk factor, *APOE* ɛ4. Locus-level analysis further confirmed AD associations of the individual alleles B*07:*02*, DRB1*15:*01*, DQA1*01:*02*, and DQB1*06:*02*. In separate class I and class II haplotype analyses, the class I A*03:01~B*07:*02* haplotype and the class II DRB1*15:01~DQA1*01:02~DQB1*06:*02* (*DR15*) haplotype were both significantly associated with risk for AD. We assessed the clinical relevance of each of these haplotypes separately in a smaller cohort representing the spectrum of cognitively normal controls and individuals with MCI and AD. Carrying the MHC class I haplotype A*03:01~B*07:*02* was associated with higher CSF amyloid levels, suggesting lower levels of amyloid in the brains of haplotype carriers across the AD spectrum. The class II haplotype *DR15* was associated with greater rate of decline on two different measures of cognitive function relevant to AD in a dose-dependent manner. In a subset of the same cohort, carrying the *DR15* risk haplotype was also associated with higher baseline levels of CC4, a biomarker of AD-related inflammation [46]. Taking these findings together, this study provides evidence for the contribution of the A*03:01~B*07:02~DRB1*15:01~DQA1*01:02~DQB1*06:*02* haplotype and its components, A*03:01~B*07:*02* and *DR15*, to risk of AD.

Over 30 y of research into HLA alleles and risk of AD has yielded mixed conclusions due in part to limitations in mapping alleles within this complicated genomic region. Early studies mapped risk of AD to the HLA region of chromosome 6 [47], and the studies that followed differed significantly in their methodological approach, the identities and resolution of the alleles studied, the ethnicity of the study cohorts, and the inferences drawn from the data. MHC class I molecule HLA-A*02 has been shown to either be associated with increased risk of AD or to have no effect in nearly 15 different studies [48–61]. Given that only B*07:*02*, DRB1*15:*01*, DQA1*01:*02*, and DQB1*06:*02* showed significant locus-level associations with AD, our findings are consistent with an ambiguous role of HLA-A*02 in AD. In terms of class II alleles, one study by Mansouri and colleagues demonstrated a link between DRB1*15:01~DQB1*06:*02* and AD in a small cohort of Tunisians [62], consistent with our findings. Previous GWASs have found that AD risk is associated with a SNP in *DRB5* [63]. As there is strong LD between DRB5*01 and DRB1*15:01~DQB1*06:*02* [64], it is possible that the AD association we have detected with *DR15* is due in part or wholly to *DRB5*. Finally, our finding that HLA associations with AD are stronger in *APOE* ɛ4-negative individuals is consistent with prior work for different HLA alleles [65,66].

The HLA region has been studied to a varying extent for its contributions to neurological disease, and many of the risk alleles implicated in the present studies have also been linked to other disorders. Most notably, the class II *DR15* haplotype is the most consistently replicated genetic finding in MS [67–69]. *DR15* also correlates with worse clinical progression in women with relapsing-onset MS (e.g., younger age at onset and more subcortical atrophy) [44]. Class I allele B*07 has also been associated with MS risk, particularly in those also carrying DRB1*15 [70]. In one Parkinson disease (PD) study, four alleles identified in a risk haplotype overlapped with our top five-allele haplotype association [71]. Similar to AD, other studies have also implicated the *HLA-DRB5* region in PD risk [72]. In one small autism study, the class I allele B*07 and class II allele DQB1*06:*02* were both associated with disease risk [73]. Finally, class II allele DQB1*06:*02* has been associated with marked increased risk for [74,75], and worsened severity of [76,77], narcolepsy. These findings are consistent with our study, in which we identified a dose-dependent association between *DR15* and greater cognitive decline in individuals representing the AD spectrum.

Participants who carried at least one copy of the class I haplotype A*03:01~B*07:*02* on average had higher baseline CSF amyloid levels, suggesting lower amyloid burden in the brains of these participants. Similar findings have been observed in *APOE* ɛ4-negative individuals when compared to *APOE* ɛ4-positive individuals across the phenotypic spectrum of cognitively normal to early MCI, suggesting higher brain amyloid β in carriers [78]. This finding raises the possibility that there could be a tau-mediated effect on AD clinical symptoms, as the AD group did not differ in clinical measures by haplotype carrier status. On the other hand, *DR15* haplotype carriers demonstrated subtle differences in baseline inflammatory biomarker levels, as well as a worse cognitive trajectory over time, suggesting a disease-modifying effect that could be mediated by changes in immune function.

Our study benefited from several strengths. The primary discovery cohort was a well-characterized sample of patients who received extensive clinical evaluations at the UCSF Memory and Aging Center. The replication dataset from the ADGC of over 11,000 AD and cognitively normal control individuals is the largest dataset to date used to explore immunogenetic contributions to AD risk. Lastly, longitudinal data from ADNI allowed us to probe the potential clinical relevance of haplotype findings across the AD spectrum. Our study also has caveats that are important to consider. Our imputation program predicted accuracy for individuals with European ancestry that was likely higher than 94.8%–99.2% based on our more stringent call threshold in comparison to other studies [38]. Imputed HLA alleles have been shown to be reliable classification tools in studies with similar methodologies [38,79,80]; however, future studies would benefit from direct sequencing of HLA alleles to avoid potential imputation inaccuracies. Because of limitations in the imputation package selected for HLA allele calling, we were only able to impute genotypes for a subset of MHC class I and II genes. For example, the imputed genes available did not include *DRB5*, which was indicated in previous studies to be associated with AD risk or pathological processes [9,24,81]. *DRB5* is on the *DR15* haplotype, so it is likely that the association we identified reflects these previous results. However, we can neither directly confirm nor refute this possibility in the present study. The *DR15* risk haplotype is most common in Europeans, and to minimize genetic heterogeneity in population substructure, we limited the present analysis to white individuals of non-Hispanic descent. Additional studies are required to assess the identified HLA risk haplotypes and component alleles for their contribution to AD in more diverse populations where patterns of LD differ and may uncouple alleles that were tightly linked in our study population, though the initial study identifying class II associations with AD in Tunisians suggests this may be a generalized risk phenomenon. Although *p* < 0.05 may be considered lenient based on the number of total alleles tested, it is also true that all of these alleles represent only five genes within one genomic region that has been previously linked to AD risk. Despite reduced statistical power due to low frequency of HLA haplotypes imparted by the extraordinary diversity of this region, we feel that this study is an important first step in elucidating the underlying contribution of the HLA to AD risk given the medical implications of ultimately identifying immune-related therapies as a means of modifying a complex, common disease. We have greater confidence in our findings due to corroborating clinical validity as identified in the ADNI cohort. Iterative subanalyses of the combined study cohort further support a role of our top five-allele haplotypes in AD risk. In addition, two of the main alleles of interest we identified, DRB1*15:*01* and DQB1*06:*02*, have been linked to AD risk in two prior studies, further supporting our results. We also identified several other risk haplotypes in our analyses beyond the ones we focused on in this study; the clinical relevance of these additional haplotypes and alleles requires further investigation. Future work is also required to test whether these findings extend to early-onset and atypical clinical syndromes with underlying AD pathology.

In summary, we present evidence for a role of the HLA class I A*03:01~B*07:*02* haplotype and the HLA class II *DR15* haplotype in AD risk. Our study also suggests that these risk haplotypes may be associated with CSF AD biomarker levels (class I) and greater decline in cognition over time, as well as higher levels of inflammation across aging (class II). The results of our study indicate that the broad A*03:01~B*07:02~DRB1*15:01~DQA1*01:02~DQB1*06:*02* haplotype may contribute genetic risk to AD beyond that contributed by the established risk factor *APOE* ɛ4, particularly in men. As components of this haplotype are well-established risk factors in MS, PD, autism, and narcolepsy, we propose that they may contribute to underlying biological risk mechanisms in multiple neurological diseases. Future work is required to establish the precise molecular processes underlying this risk association, as well as to expand this finding to broader, diverse populations of AD and potentially even other neurodegenerative conditions.

## Supporting information

S1 Acknowledgments Detailed acknowledgments for ADNI and ADGC and other samples used in this study. (PDF)

S1 Fig Box-and-whisker plot of posterior probabilities of imputation for each of five imputed HLA-alleles in the ADGC cohort. The thick line represents the median, box edges represent the first and third quartiles, and whiskers represent the 95% CI. Values higher than 0.75 (dashed line) were included in the present study. Outlier dots are not shown for clarity.
(DOCX)

S2 Fig Box-and-whisker plots of AD cognitive and clinical biomarker measures in HLA A*03:01~B*07:*02* haplotype noncarriers and carriers in the ADNI cohort. HLA A*03:01~B*07:*02* haplotype carriers (*n* = 3) do not show any significant differences from haplotype noncarriers (*n* = 67) in a variety of cognitive assessments and measures of biomarkers in CSF in the ADNI cohort. The thick line represents the median, box edges represent the first and third quartiles, and whiskers represent the 95% CI. MMSE, Mini Mental State Exam; p-tau, phosphorylated tau.
(DOCX)

S3 Fig Box-and-whisker plots of AD cognitive and clinical biomarker measures in *DR15* haplotype noncarriers and carriers in the ADNI cohort. *DR15* haplotype carriers (*n* = 23) do not show any significant differences from haplotype noncarriers (*n* = 47) in a variety of cognitive assessments and measures of biomarkers in CSF in the ADNI cohort. The thick line represents the median, box edges represent the first and third quartiles, and whiskers represent the 95% CI. MMSE, Mini Mental State Exam; p-tau, phosphorylated tau.
(DOCX)

S4 Fig Chemokine CC4 levels are higher in older adults. Chemokine CC4 levels are on average higher with greater age, suggesting higher levels of inflammation in older individuals. Chemokine CC4 levels are quality controlled and transformed as described in S1 Methods. The plotted points are partial residuals with 95% confidence bands provided in shading.
(DOCX)

S1 File Analysis plan. (DOCX)

S1 List List of immune and inflammation-related CSF biomarkers included in the analysis. (DOCX)

S1 Methods ADNI diagnosis and CSF biomarker information. (PDF)

S1 Table Summary information for datasets that make up the ADGC merged dataset. Study name, male/female distribution, case/control/missing distribution, and sample size of 30 datasets containing unrelated individuals combined into the full ADGC dataset.
(DOCX)

S2 Table Summary statistics for ADNI participants with baseline CSF protein measurements. Descriptive data are summarized by diagnostic category. Values represent the mean ± standard error, percent, or number of participants in a given diagnostic category. Two-tailed *p*-values were from ANOVA (continuous traits) or chi-square (categorical values) tests by diagnostic group. CN, cognitively normal; NS, not significant (*p* > 0.05).
(DOCX)

S3 Table HLA A*03:01~B*07:*02* haplotype carriers do not show any significant baseline differences on clinical biomarker measures of AD with the exception of CSF amyloid β level. Analysis of patients with baseline AD diagnosis from the ADNI cohort carrying HLA A*03:01~B*07:*02* (*n* = 3) versus haplotype noncarriers (*n* = 63) shows no significant differences in volumetric, clinical, cognitive, and biomarker assessments relevant to AD with the exception of amyloid β levels as measured in CSF (*p* = 0.03). The *p*-values for volumetric measurements are the effect of carrying the DR15 haplotype (binary 0/1) in a linear regression model adjusted for baseline age, sex, years of education, dose of *APOE* ε4 allele (0/1/2), and intracranial volume. The *p*-values for clinical and biomarker measures are the effect of carrying the DR15 haplotype (binary 0/1) in a linear regression model adjusted for baseline age, sex, years of education, and dose of *APOE* ε4 allele (0/1/2). ADAS11, 11-item Alzheimer’s Disease Assessment Scale cognitive subscale; MMSE, Mini-Mental State Exam.
(DOCX)

S4 Table HLA *DR15* risk haplotype dosage is associated with longitudinal changes in ADAS and RAVLT cognitive test scores. Results from regression models used to determine the effect of dose of HLA risk haplotype DRB1*15:01~DQA1*01:02~DQB1*06:*02* on longitudinal changes in ADAS and RAVLT cognitive test scores in cognitively normal, MCI, and AD groups from the ADNI cohort. The beta estimate (Estimate) and accompanying standard error (SE) reflect the adjusted effect of each independent variable as a predictor of ADAS 11-item score and RAVLT forgetting index scores. HLA haplotype dose demonstrated a significant positive and negative association with the rate of change in the ADAS (*p* = 0.02) and RAVLT (*p* = 0.03) scores, respectively, across all diagnostic groups (Time x Haplotype Dose). In other words, a greater dose of risk haplotype was associated with worse decline in cognitive performance over time. For all disease groups, the linear statistical model included the following as independent variables: age, time (from baseline), sex, CDR-SB score, *APOE ε*4 carrier status, education, and haplotype dose. All tests were two-tailed.
(DOCX)

S5 Table *DR15* haplotype carriers do not show any significant baseline differences on clinical biomarker measures of AD. Analysis of patients with baseline AD diagnosis from the ADNI cohort carrying DR15 (*n* = 23) versus DR15 noncarriers (*n* = 47) show no significant differences in volumetric, clinical, cognitive, and biomarker assessments relevant to AD. The *p*-values for volumetric measurements are the effect of carrying the DR15 haplotype (binary 0/1) in a linear regression model adjusted for baseline age, sex, years of education, dose of *APOE* ε4 allele (0/1/2), and intracranial volume. The *p*-values for clinical and biomarker measures are the effect of carrying the DR15 haplotype (binary 0/1) in a linear regression model adjusted for baseline age, sex, years of education, and dose of APOE ε4 allele (0/1/2). MMSE, Mini-Mental State Exam.
(DOCX)

S6 Table HLA *DR15* risk haplotype dosage is associated with baseline levels of chemokine CC-4 in CSF. Regression models were used to determine the effect of HLA risk haplotype DRB1*15:01~DQA1*01:02~DQB1*06:*02* dose on cross-sectional CSF levels of chemokine CC-4 in the cognitively normal, MCI, and AD groups of the ADNI cohort are summarized. The beta estimate (Estimate) and accompanying standard error (SE) reflect the adjusted effect of each independent variable as a predictor of chemokine CC-4 levels. HLA risk haplotype dose demonstrated a significant positive association with baseline chemokine CC-4 CSF levels across all diagnostic groups (Haplotype Dose, *p* = 5.18 x 10^−3^) such that more copies of the risk haplotype were associated with higher levels of CSF chemokine CC-4, a measure of inflammation. For all disease groups, the linear statistical model included the following as independent variables: age, sex, CDR-SB score, *APOE ε*4 carrier status, education, and haplotype dose. All tests were two-tailed.
(DOCX)

S7 Table Five-allele haplotypes with significant risk associations in individual sexes. All significant five-allele haplotype results (*p < 0.05) for the combined UCSF + ADGC cohort (*n* = 11,690) when males and females are analyzed separately. Two of the three most significant five-allele haplotypes from the combined analysis (males + females) were significant in an individual sex analysis and are highlighted in this table in bold. Nonsignificant results are shown in grey. In addition to the OR with the 95% CI, a breakdown of haplotype frequency in individuals with AD versus cognitively normal older adult controls is also provided.
(DOCX)

S8 Table Class I and class II haplotypes with significant risk associations in individual sexes. All significant class I (two-allele) and class II (three-allele) haplotype results (*p < 0.05) for the combined UCSF + ADGC cohort (*n* = 11,690) when males and females are analyzed separately. Class I and class II haplotypes present in one of the three most significant five-allele from the combined analysis (males + females) are highlighted in this table in bold. Nonsignificant results are shown in grey. In addition to OR with 95% CI, a breakdown of haplotype frequency in individuals with AD versus cognitively normal older adult controls is also provided.
(DOCX)

S9 Table Individual alleles with significant risk associations in individual sexes. All significant loci results (*p < 0.05) for combined UCSF + ADGC cohort (*n* = 11,690) when males and females are analyzed separately. Alleles present in one of the top three most significant five-allele haplotypes from the combined analysis (A*02:01~B*13:02~DRB1*07:01~DQA1*02:01~DQB1*02:*02*, A*03:01~B*07:02~DRB1*15:01~DQA1*01:02~DQB1*06:*02* and A*24:02~B*38:01~DRB1*13:01~DQA1*01:03~DQB1*06:*03*) are shown in this table in bold. Nonsignificant results are shown in grey. In addition to OR with 95% CI, a breakdown of allele frequency in individuals with AD versus cognitively normal older adult controls is also provided.
(DOCX)

S10 Table Twenty iterative analyses of the randomly split ADGC + UCSF combined cohort to corroborate top five-allele haplotype associations with AD risk. We randomly split our full ADGC + UCSF cohort (*n* = 11,381) in half ten times, balancing AD and cognitively normal controls, to determine how often the haplotypes we found to be significant (*p* < 0.05) in our original analysis replicated in these 20 smaller cohort analyses. The OR, 95% CI, and p-value from the original analysis (values from Table 2) are listed for each haplotype, and results are listed for any of the replication cohorts in which a given haplotype was significant. Each haplotype also lists the number of smaller cohorts in which a significant finding was found (n rep) and the percentage (number of replication cohorts / 20). In these 20 analyses, two of our top-associated five-allele haplotypes, A*03:01~B*07:02~DRB1*15:01~DQA1*01:02~DQB1*06:*02* and A*02:01~B*13:02~DRB1*07:01~DQA1*02:01~DQB1*02:*02*, showed up as significant in over half of the smaller analyses, further corroborating their role in AD risk.
(DOCX)

## Abbreviations

AD: Alzheimer disease
ADAS: Alzheimer’s Disease Assessment Scale
ADAS11: 11-item Alzheimer’s Disease Assessment Scale cognitive subscale
ADGC: Alzheimer’s Disease Genetics Consortium
ADNI: Alzheimer’s Disease Neuroimaging Initiative
APOE: apolipoprotein E
APP: amyloid precursor protein
CDR-SB: Clinical Dementia Rating sum of boxes
CI: confidence interval
CN: cognitively normal
CSF: cerebrospinal fluid
GWAS: genome-wide association study
HLA: human leukocyte antigen
LD: linkage disequilibrium
MAC: Memory and Aging Center
MCI: mild cognitive impairment
MHC: major histocompatibility complex
MS: multiple sclerosis
NIA: National Institute on Aging
OR: odds ratio
PD: Parkinson disease
PSEN1: presenilin 1
PSEN2: presenilin 2
RAVLT: Rey Auditory Verbal Learning Test
SNP: single nucleotide polymorphism
UCSF: University of California, San Francisco
