# Supplementary material for: Fine-mapping of the human leukocyte antigen locus as a risk factor for Alzheimer disease: A case–control study
Source: PLoS Med. 2017 Mar 28;14(3):e1002272. doi: 10.1371/journal.pmed.1002272 (PMC5369701; doi:10.1371/journal.pmed.1002272)

**S1 Fig. Box and whiskers plot of posterior probabilities of imputation for each of 5 imputed Human Leukocyte Antigen alleles in the Alzheimer’s Disease Genetics Consortium cohort.** Thick line represents median, box edges represent 1^st^ and 3^rd^ quartile, and whiskers represent 95% confidence interval. Values higher than 0.75 (dashed line) were included in the present study. Outlier dots are not shown for clarity**.**


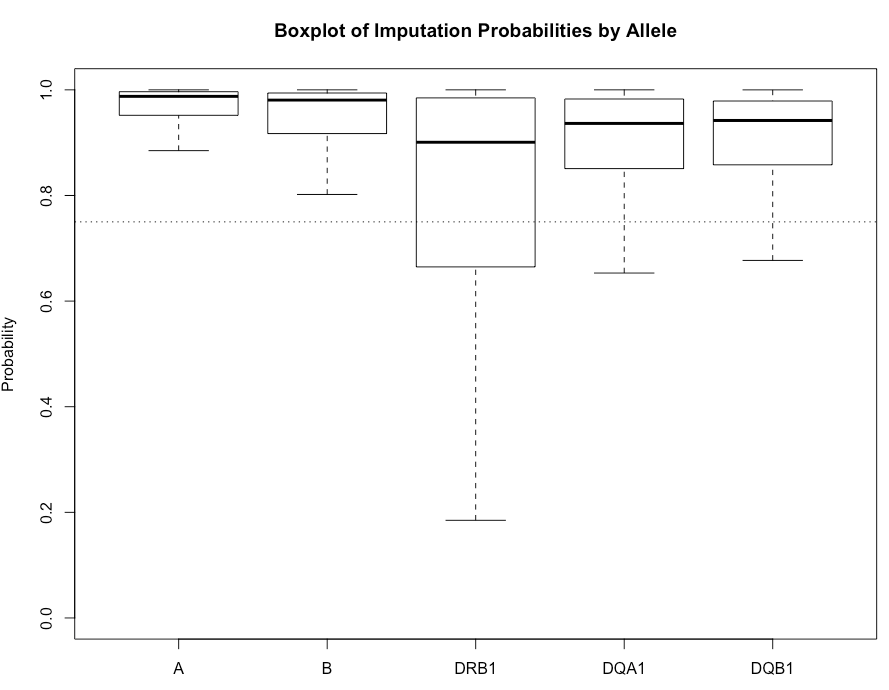

Supplement: S1 Fig — The thick line represents the median, box edges represent the first and third quartiles, and whiskers represent the 95% CI. Values higher than 0.75 (dashed line) were included in the present study. Outlier dots are not shown for clarity. (DOCX) [file pmed.1002272.s002.docx]
