# Supplementary material for: Fine-mapping of the human leukocyte antigen locus as a risk factor for Alzheimer disease: A case–control study
Source: PLoS Med. 2017 Mar 28;14(3):e1002272. doi: 10.1371/journal.pmed.1002272 (PMC5369701; doi:10.1371/journal.pmed.1002272)

**S2 Fig: Box-and-whisker plots of Alzheimer’s Disease cognitive and clinical biomarker measures in HLA-A*03:01~HLA-B*07:02 haplotype non-carriers and carriers in Alzheimer’s Disease Neuroimaging Initiative (ADNI) cohort.** HLA-A*03:01~HLA-B*07:02 haplotype carriers (n = 3) do not show any significant differences from haplotype non-carriers (n = 67) in a variety of cognitive assessments and measures of biomarkers in cerebrospinal fluid (CSF) in the ADNI cohort. Thick line represents median, box edges represent 1^st^ and 3^rd^ quartile, and whiskers represent 95% confidence interval. CDR-SB = Clinical Dementia Rating Sum of Boxes, MMSE = Mini Mental State Exam, ADAS = Alzheimer’s Disease Assessment Scale, RAVLT = Rey Auditory Verbal Learning Test, CSF = cerebrospinal fluid, p-tau = phosphorylated tau.


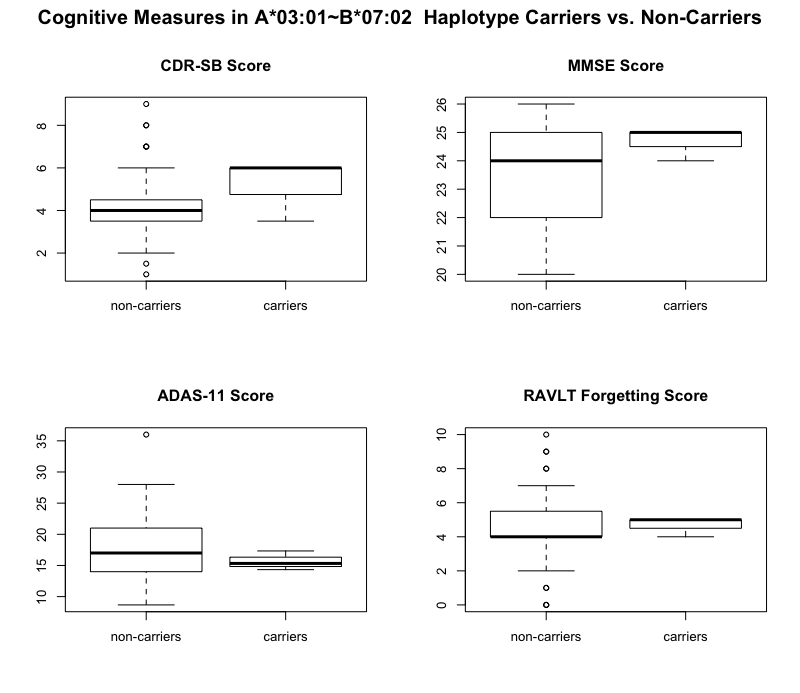


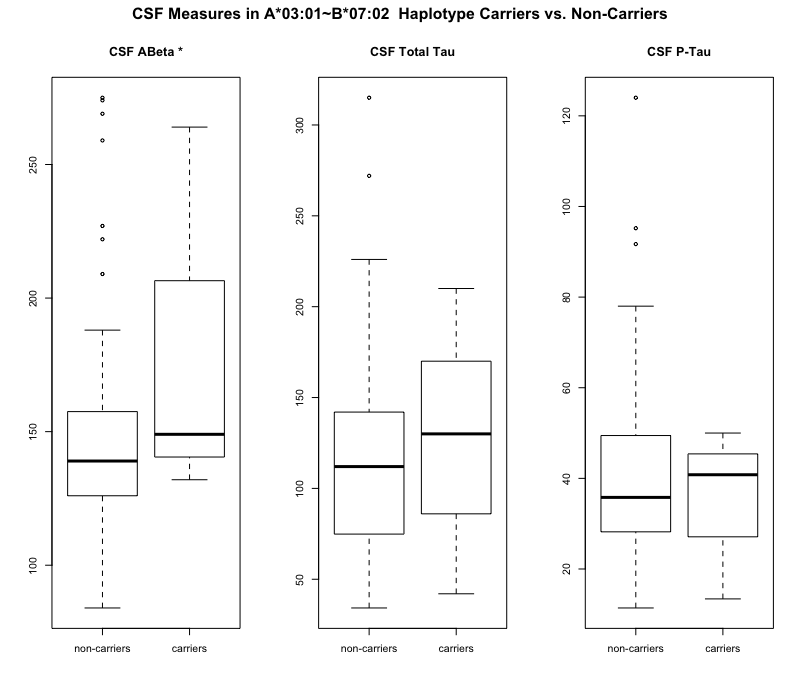

Supplement: S2 Fig — HLA A*03:01~B*07:02 haplotype carriers (n = 3) do not show any significant differences from haplotype noncarriers (n = 67) in a variety of cognitive assessments and measures of biomarkers in CSF in the ADNI cohort. The thick line represents the median, box edges represent the first and third quartiles, and whiskers represent the 95% CI. MMSE, Mini Mental State Exam; p-tau, phosphorylated tau. (DOCX) [file pmed.1002272.s003.docx]
