# Supplementary material for: Fine-mapping of the human leukocyte antigen locus as a risk factor for Alzheimer disease: A case–control study
Source: PLoS Med. 2017 Mar 28;14(3):e1002272. doi: 10.1371/journal.pmed.1002272 (PMC5369701; doi:10.1371/journal.pmed.1002272)

**S4 Fig. Chemokine CC4 levels are higher in older adults.** Chemokine CC4 levels are on average higher with greater age, suggesting higher levels of inflammation in older individuals. Chemokine CC4 levels are quality controlled and transformed as described in Supplemental Methods. The plotted points are partial residuals with 95% confidence bands provided in shading.


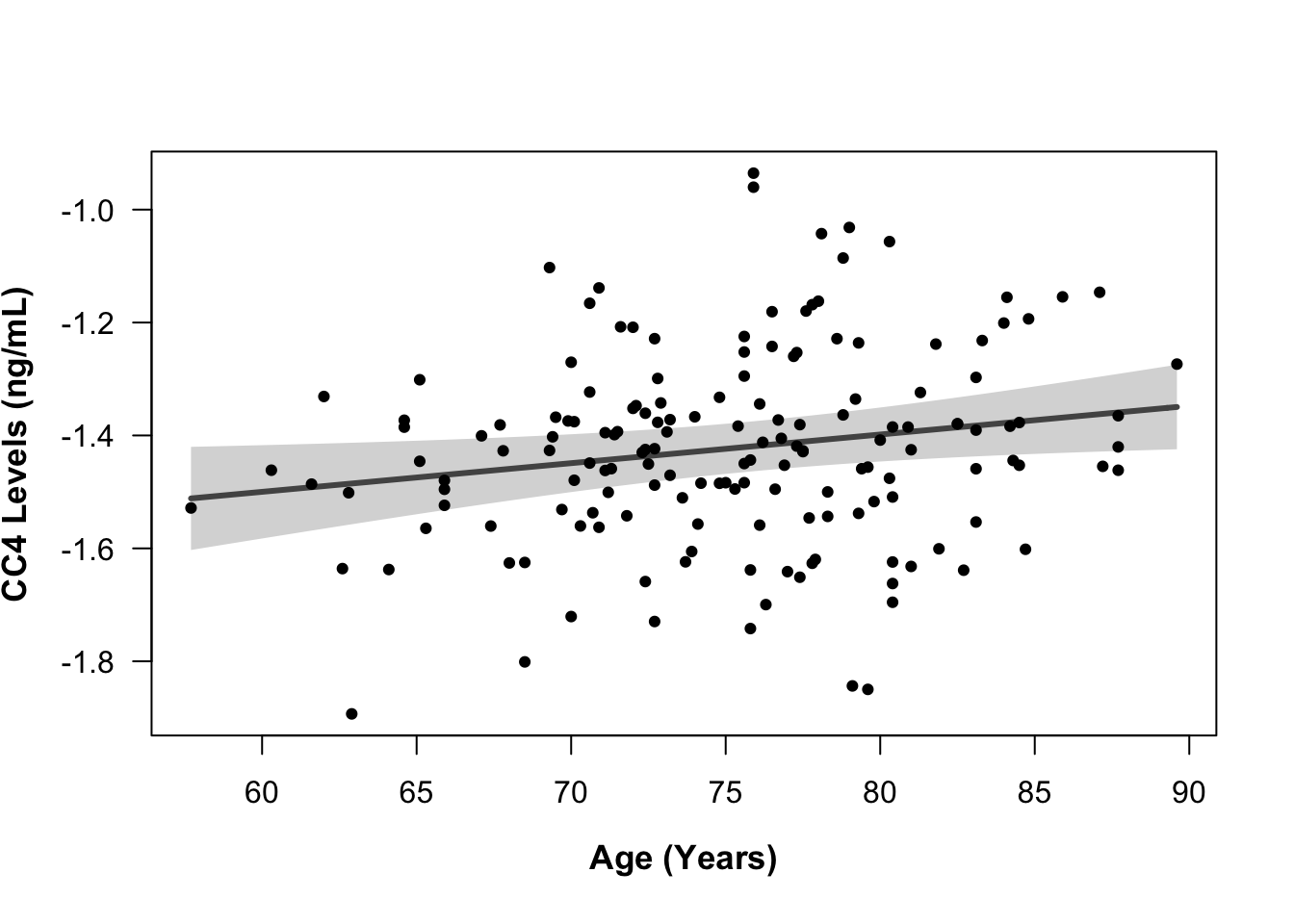

Supplement: S4 Fig — Chemokine CC4 levels are on average higher with greater age, suggesting higher levels of inflammation in older individuals. Chemokine CC4 levels are quality controlled and transformed as described in S1 Methods. The plotted points are partial residuals with 95% confidence bands provided in shading. (DOCX) [file pmed.1002272.s005.docx]
