# Supplementary material for: Fine-mapping of the human leukocyte antigen locus as a risk factor for Alzheimer disease: A case–control study
Source: PLoS Med. 2017 Mar 28;14(3):e1002272. doi: 10.1371/journal.pmed.1002272 (PMC5369701; doi:10.1371/journal.pmed.1002272)
Supplement: S1 File — (DOCX) [file pmed.1002272.s006.docx]

**S1 File: Analysis proposal to examine HLA allelic/haplotype associations with risk of Alzheimer’s disease in exploratory UCSF cohort analysis and Alzheimer’s Disease Genetics Consortium replication dataset**

The proposal below is our original submission request to obtain data from the Alzheimer’s Disease Genetics Consortium (ADGC). This study began as a fine-mapping investigation to follow up a recently published GWAS-based pleiotropy analysis by our group that showed a locus near HLA-DRB5 significantly associated with susceptibility to AD and diseases with an autoimmune component. As this was an attempt to fine map the DRB5 signal observed, we set our significance threshold at p<0.05 at the locus level. Following our analyses of UCSF data, we attempted to replicate our results in independent cohorts (ADGC). An additional benefit of these cohorts was the availability of cognitive and biomarker data in a subset of individuals (ADNI, which were not included in the primary association analysis but were instead used as a 3^rd^ independent cohort for analysis); this allowed assessment of clinical validity of significant association findings to further contextualize our results.

**Investigation OF HLA CONTRIBUTIONS TO RISK FOR Alzheimer’s disease**

**Principal Investigator:** Jennifer S. Yokoyama, PhD, University of California, San Francisco

**Other Investigators:** Natasha Rabinowitz Steele, MPH, Jill Hollenbach, PhD, MPH, Vincent Damotte, PhD, Luke Bonham, Ethan Geier, PhD, Bruce Miller, MD

**Rationale:**

The human leukocyte antigen (HLA) system encodes the major histocompatibility complex (MHC) cell surface proteins that play a major role in regulation of the immune system. There is increasing recognition that immune regulation and related inflammatory pathways play a complex role in the etiology of different neurodegenerative diseases.

Previous GWAS studies and meta-analyses by our group and others have investigated MHC

susceptibility loci in a wide range of diseases, including Alzheimer’s Disease (AD)[1,2]

However, due to the comple genetic organization of the HLA region, studies have yet to

elucidate which genes and specific alleles contribute to the signal observed.

We seek to further characterize genetic risks for errors in immune regulation and their implication for the pathobiology of AD. Using data from the literature on other neurodegenerative diseases such as multiple sclerosis (MS) and Parkinson’s disease (PD), we will also explore the overlap of specific immunogenetic risk factors associated with AD within the HLA region with other established risk loci.

**Hypothesis:**

We hypothesize that there are HLA alleles and haplotypes that confer risk to late-onset AD, as well as HLA alleles associated with healthy aging phenotypes.

**Research Questions:**

1. Can HLA haplotype, specific alleles, or amino acid analysis predict risk for AD when compared to healthy older controls?
2. Do HLA genetic risk factors associated with AD overlap with those of other neurodegenerative diseases, i.e.; MS or PD?

**Datasets:** genome-wide SNP data from ADC, ADNI, ROS. Preprocessed GWAS data is sufficient for our proposed analysis.

**Pending affirmation for collaboration and confirmation of necessary data.*

**GWAS data for AD:** The table below lists the GWAS datasets relevant to AD that may be available to approved investigators. Datasets will be added as they become available.

| Cohort | Cases | Controls | Platform | Total Subjects |
| --- | --- | --- | --- | --- |
| ACT/eMERGE*^,1^ | 566 | 1,696 | Illumina 660Quad | 2,262 |
| ADC | 3,437 | 1,618 | Illumina 660Quad, Illumina  Omni Express | 5,055 |
| ADNI^2^ | 268 | 173 | Illumina 610Quad | 441 |
| GSK^3^ | 655 | 641 | Affymetrix 500K | 1,296 |
| UM/VU/MSSM*^,4^ | 1,186 | 1,135 | Illumina 550, 610Quad, 1M,  1M-duo; Affymetrix 6.0 | 2,321 |
| MIRAGE^*,5^ | 603 | 885 | Illumina 660Quad | 1,488 |
| NIA LOAD^*,6^ | 1,839 | 1,983 | Illumina 610Quad | 3,822 |
| OHSU*^,7^ | 380 | 133 | Illumina 370K | 513 |
| TGEN-2*^,8^ | 670 | 365 | Affymetrix 600K | 1,035 |
| Mayo^*,9^ | 844 | 1,255 | Illumina 300K | 2,099 |
| ROS^*,10^ | 495 | 1,153 | Affymetrix 6.0 | 1,648 |
| UPitt^*,11^ | 1,400 | 1,000 | 1M Omni | 2,400 |
| Total | 12,343 | 12,037 |  | 24,380 |

* Need to consult with the PIs on these projects to obtain permission to use data:

1. Eric Larson

2. Andrew Saykin

3. Peter H. St. George-Hyslop

4. Joseph Buxbaum, Jonathan Haines, and Margaret Pericak-Vance

5. Lindsay Farrer

6. Richard Mayeux

7. Patricia Kramer

8. Eric Reiman

9. Steve Younkin

10. David Bennett

11. Ilyas Kamboh

**Additional data:**

**Predictor Variables:** (1) imputed HLA alleles, (2) imputed HLA haplotypes, (3) HLA amino acid positions that define specific HLA alleles

**Outcome Variable:** 1. Disease status (AD, control)

**Other covariates of interest:** If available, we will explore binned analyses separating groups of patients and controls based on *APOE* e4 carrier status

**Analysis (all to be performed by J. Yokoyama):**

*Genetic Analyses.* As the largest majority of ADC, ADNI, and ROS cohorts’ genotyped samples are from white participants, all genetic analyses will be performed in whites only. This study design allows for maximum statistical power for replication of primary findings across each independent cohort and reduces potential for confounding due to the known population-based contribution to diversity in the HLA region. Despite limiting our genetic analyses to whites, it is still prudent to perform assessment for population sub-structure within all sample cohorts as subtle differences in ancestry (e.g., Hispanic versus non-Hispanic) will also have the potential to confound genetic association analyses. Ten multi-dimensional scaling (MDS) covariates will be calculated utilizing an unlinked (r^2^ < 0.2) subset of genome-wide SNPs for all samples in PLINK [3]. MDS vectors will be calculated within each cohort and only on SNPs that have been directly genotyped (in cohorts with imputed data). We may also adjust all analyses by demographic factors including age and sex if they are determined to be confounders in the assessment of AD risk in these cohorts.

**Methodology:**

a. Derive HLA genotypes from SNP data using an imputation program that makes predictions by averaging HLA-type posterior probabilities over an ensemble of classifiers built on bootstrap samples.  We will run primary imputation in HIBAG [4] and will explore alternative imputation programs to assess accuracy and genotype concordance across programs.

b. Conduct analysis of AD patients and healthy controls using BIGDAWG, a statistical package in R designed to specifically probe associations with the HLA [5], including tests of Hardy-Weinberg equilibrium, case-control association analyses for haplotypes, individual loci, and HLA amino acid positions. Analyses will be performed for each cohort separately due to differences in genotyping platform that may affect imputation of HLA alleles. Each cohort will be assessed as an independent cohort with the goal of replicating preliminary findings we have already obtained from analysis of our own center’s genetic data from late-onset AD patients and healthy older controls.

c. We will compare AD-associated HLA alleles, haplotypes, and/or amino acids to those previously implicated in other neurodegenerative diseases such as MS and PD and assess for overlap across disease groups. If similar alleles have been implicated in multiple diseases, we may combine results via meta-analysis using other MS and PD genetic datasets.

**Timeline:**

*Summer 2016:* submit proposal to ADGC; obtain affirmation from additional collaborators, data acquisition and QC, imputation processes, and primary analyses.

*Fall 2016:* prepare manuscript and submit to ADGC for approval

*Winter 2016:* submit abstract to ADGC for approval and present at conference

**Deliverables:** manuscript describing HLA haplotypes, specific loci, and amino acids associated with risk for AD and their relationship to risk loci associated with other neurodegenerative disorders.

Thank you for considering this proposal.

Jennifer S. Yokoyama

References:

1. Lambert JC, Ibrahim-Verbaas CA, Harold D, Naj AC, Sims R, Bellenguez C, et al. Meta-analysis of 74,046 individuals identifies 11 new susceptibility loci for Alzheimer’s disease. Nat Genet. 2013;45: 1452–1458. doi:10.1038/ng.2802

2. Yokoyama JS, Wang Y, Schork AJ, Thompson WK, Karch CM, Cruchaga C, et al. Association between genetic traits for immune-mediated diseases and Alzheimer disease. JAMA Neurol. 2016;73. doi:10.1001/jamaneurol.2016.0150

3. Purcell S, Neale B, Todd-Brown K, Thomas L, Ferreira MAR, Bender D, et al. PLINK: a tool set for whole-genome association and population-based linkage analyses. Am J Hum Genet. 2007;81: 559–575.

4. Khor S-S, Yang W, Kawashima M, Kamitsuji S, Zheng X, Nishida N, et al. High-accuracy imputation for HLA class I and II genes based on high-resolution SNP data of population-specific references. Pharmacogenomics J. 2015;15: 530–7. doi:10.1038/tpj.2015.4

5. Pappas DJ, Marin W, Hollenbach JA, Mack SJ. Bridging ImmunoGenomic Data Analysis Workflow Gaps (BIGDAWG): An integrated case-control analysis pipeline. Hum Immunol. 2016;77: 283–287. doi:10.1016/j.humimm.2015.12.006
