# Supplementary material for: Fine-mapping of the human leukocyte antigen locus as a risk factor for Alzheimer disease: A case–control study
Source: PLoS Med. 2017 Mar 28;14(3):e1002272. doi: 10.1371/journal.pmed.1002272 (PMC5369701; doi:10.1371/journal.pmed.1002272)
Supplement: S1 List — (DOCX) [file pmed.1002272.s007.docx]

**S1 List. List of immune and inflammation-related cerebrospinal fluid biomarkers included in analysis**

| **Analyte** |
| --- |
| Beta 2 Microglobulin |
| C-Reactive Protein |
| CD 40 antigen |
| Chemokine CC-4 |
| Complement C3 |
| Cortisol |
| Fas Ligand |
| Immunoglobulin A |
| Intercellular Adhesion Molecule 1 |
| Interferon gamma Induced Protein 10 |
| Interleukin-16 |
| Interleukin-25 |
| Interleukin-3 |
| Interleukin-6 receptor |
| Interleukin-8 |
| Macrophage Colony-Stimulating Factor 1 |
| Macrophage Inflammatory Protein-1 beta |
| Macrophage Migration Inhibitory Factor |
| Monocyte Chemotactic Protein 1 |
| Monocyte Chemotactic Protein 2 |
| Monokine Induced by Gamma Interferon |
| Neutrophil Gelatinase-Associated Lipocalin |
| Osteopontin |
| Resistin |
| Stem Cell Factor |
| T Lymphocyte-Secreted Protein I-309 |
| T-Cell-Specific Protein RANTES |
| Thrombomodulin |
