# Supplementary material for: Fine-mapping of the human leukocyte antigen locus as a risk factor for Alzheimer disease: A case–control study
Source: PLoS Med. 2017 Mar 28;14(3):e1002272. doi: 10.1371/journal.pmed.1002272 (PMC5369701; doi:10.1371/journal.pmed.1002272)
Supplement: S1 Methods — (PDF) [file pmed.1002272.s008.pdf]

S1 Methods. ADNI Diagnosis and CSF Biomarker Information

ADNI Diagnostic Guidelines

Controls were required to have normal memory function on the Logical Memory II subscale of the Weschler Memory Scale– Revised (Binder, Storandt, & Birge, 1999), an MMSE score greater than 24, CDR total score equal to 0, and clinical determination that the individual was not significantly impaired in cognitive function or activities of daily living. Individuals with MCI were required to have abnormal memory function on the Logical Memory II subscale of the Weschler Memory Scale – Revised, an MMSE greater than 24, CDR total score equal to 0.5, and clinical determination that the individual's general cognition and functional performance was impaired enough to make a diagnosis of AD. Finally, individuals with AD were required to have abnormal memory function on the Logical Memory II subscale of the Weschler Memory Scale – Revised, an MMSE between 20 and 26, CDR total score equal to 0.5 or 1.0, and judgment by a clinician that the individual met NINCDS/ADRDA criteria for probable AD (Mckhann et al., 2011).

ADNI CSF Biomarker Measurement

Baseline CSF CC4 levels were measured using the Human DiscoveryMAP panel developed by Rules Based Medicine (Myriad RBM; Austin, Texas). The Human DiscoveryMAP panel is commercially available and measures a collection of metabolic, lipid, inflammatory, and other AD-relevant indicators. A full list of the measured metabolites is available through Myriad RBM. The CSF measurements in the immunoassay panel were processed and normalized according to previously described methods (Craig-Schapiro et al., 2011; Siuciak, 2011). Briefly, Myriad RBM used a Luminex 100 instrument for the measurements and analyzed the resulting data using proprietary software. The ADNI staff checked analyte distributions for normality using Box-Cox analyses and, if needed, log10 transformed the data to achieve an approximately normal distribution. Out of 83 biomarkers with sufficient data available for analysis, we selected 28 that were most directly relevant to immune function and inflammation (S1 List).

References

Binder, E. F., Storandt, M., & Birge, S. J. (1999). The Relation Between Psychometric Test Performance and Physical Performance in Older Adults. *The Journals of Gerontology Series A: Biological Sciences and Medical Sciences*, *54*(8), M428–M432. https://doi.org/10.1093/gerona/54.8.M428

Craig-Schapiro, R., Kuhn, M., Xiong, C., Pickering, E. H., Liu, J., Misko, T. P., … Holtzman, D. M. (2011). Multiplexed immunoassay panel identifies novel CSF biomarkers for alzheimer's disease diagnosis and prognosis. *PLoS ONE*, *6*(4). https://doi.org/10.1371/journal.pone.0018850

Mckhann, G., Knopman, D. S., Chertkow, H., Hyman, B. T., Jack, C. R., Kawas, C., … Phelps, C. (2011). The diagnosis of dementia due to Alzheimer's disease: Recommendations from the National Institute on Aging-Alzheimer's Association workgroups on diagnostic guidelines for Alzheimer's disease. *Alzheimer's and Dementia*, *7*(3), 263–269. JOUR. Retrieved from file:///Users/jsy/Dropbox/Papers/2011/Mckhann/Mckhann2011_Alzheimer's and Dementia.pdf

Siuciak J. Biomarkers Consortium Data Primer. 2011
