# Supplementary material for: Fine-mapping of the human leukocyte antigen locus as a risk factor for Alzheimer disease: A case–control study
Source: PLoS Med. 2017 Mar 28;14(3):e1002272. doi: 10.1371/journal.pmed.1002272 (PMC5369701; doi:10.1371/journal.pmed.1002272)
Supplement: S1 Table — Study name, male/female distribution, case/control/missing distribution, and sample size of 30 datasets containing unrelated individuals combined into the full ADGC dataset. (DOCX) [file pmed.1002272.s009.docx]

**S1 Table. Summary information for datasets that make up Alzheimer’s Disease Genetics Consortium (ADGC) merged dataset.** Study name, male (M) / female (F) distribution, case/control/missing distribution, and sample size of 30 datasets containing unrelated individuals combined into full ADGC dataset.

| **Study** | **Sex(M/F)** | **Cases/Controls/Missing** | **Sample Size** |
| --- | --- | --- | --- |
| ACT 1 | 886/1,161 | 479/1,348/220 | 2,047 |
| ADC 1 | 947/1,137 | 1,503/543/38 | 2,084 |
| ADC 2 | 328/364 | 546/121/25 | 692 |
| ADC 3 | 593/721 | 711/464/139 | 1,314 |
| ADNI | 317/207 | 215/140/169 | 524 |
| GSK/GenADA | 608/952 | 796/764/0 | 1,560 |
| LOAD/NIA-LOAD | 628/1,069 | 745/801/151 | 1,697 |
| YOUNKIN/MAYO | 706/835 | 616/925/0 | 1,541 |
| MIRAGE | 274/429 | 398/294/13 | 705 |
| KRAMER/OHSU | 142/188 | 59/109/162 | 330 |
| ROSMAP | 502/1,119 | 364/853/404 | 1,621 |
| TGEN2 | 560/698 | 770/488/0 | 1,258 |
| MIAMI/UMVUMSSM | 817/1,380 | 1,085/1,112/0 | 2,197 |
| KAMBOH2/UPITT | 810/1,377 | 1,267/834/86 | 2,187 |
| WASHU/GOATE | 217/295 | 312/166/34 | 512 |
| ACT 2 | 144/158 | 18/5/279 | 302 |
| ADC 4 | 332/443 | 287/340/148 | 775 |
| ADC 5 | 370/526 | 273/496/127 | 896 |
| ADC 6 | 432/571 | 363/304/336 | 1,003 |
| BIOCARD | 75/113 | 8/123/57 | 188 |
| CHAP | 236/348 | 20/164/400 | 584 |
| EAS | 116/132 | 10/209/29 | 248 |
| MTV | 177/261 | 241/194/3 | 438 |
| NBB | 96/204 | 215/85/0 | 300 |
| RMAYO | 220/133 | 12/271/70 | 353 |
| ROSMAP 2 | 105/323 | 62/237/129 | 428 |
| TARC1 | 170/260 | 286/144/1 | 431 |
| UKS | 845/895 | 767/973/0 | 1,740 |
| WASHU 2 | 68/67 | 30/65/40 | 135 |
| WHICAP | 246/394 | 74/562/4 | 640 |
| Total (Combined Phase 1 and 2) | 11,967/16,760 | 12,532/13,134/3,064 | 28,730 |

**Table reproduced from:**

Boehme KL, Mukherjee S, Crane PK, Kauwe JSK. ADGC 1000 genomes combined workflow (electronic document). September 2014. Accessible at: <https://kauwelab.byu.edu/ADGC1KGImputation.aspx>

**ROSMAP References:**

Bennett DA, Schneider JA, Arvanitakis Z, Wilson RS. Overview and findings from the Religious Orders Study. *Current Alzheimer’s Research*. 2012;9:628-645. PMCID: PMC3409291

Bennett DA, Schneider JA, Buchman AS, Barnes LL, Boyle PA, Wilson RS. Overview and findings from the Rush Memory and Aging Project. *Current Alzheimer’s Research*. 2012;9:646-663.
