# Supplementary material for: Fine-mapping of the human leukocyte antigen locus as a risk factor for Alzheimer disease: A case–control study
Source: PLoS Med. 2017 Mar 28;14(3):e1002272. doi: 10.1371/journal.pmed.1002272 (PMC5369701; doi:10.1371/journal.pmed.1002272)
Supplement: S2 Table — Descriptive data are summarized by diagnostic category. Values represent the mean ± standard error, percent, or number of participants in a given diagnostic category. Two-tailed p-values were from ANOVA (continuous traits) or chi-square (categorical values) tests by diagnostic group. CN, cognitively normal; NS, not significant (p > 0.05). (DOCX) [file pmed.1002272.s010.docx]

**S2 Table.**

|  | CN | MCI | AD | *P*-Value |
| --- | --- | --- | --- | --- |
| N | 49 | 61 | 53 |  |
| Age (years) | 75.6 ± 5.41 | 74.3 ± 6.59 | 75.8 ± 6.56 | NS |
| Female (%) | 57.1% | 29.5% | 47.2% | < 0.05 |
| Education (years) | 15.5 ± 2.54 | 15.3 ± 3.29 | 14.6 ± 3.12 | NS |
| CDR-SB | 0.14 ± 0.14 | 1.66 ± 0.89 | 4.18 ± 1.67 | < 0.001 |
| *APOE* ε4 Carrier (%) | 30.6% | 60.7% | 71.7% | < 0.001 |
| Haplotype Dose  (# single / # double) | 20/1 | 15/0 | 20/1 | NS |
| Chemokine CC-4 (ng/mL) | -1.47 ± 0.17 | -1.41 ± 0.20 | -1.49 ± 0.20 | NS |
| ADAS (baseline) | 5.86 ± 2.78 | 12.3 ± 3.41 | 17.9 ± 5.49 | < 0.001 |
| RAVLT Forgetting (baseline) | 4.08 ± 3.02 | 5.13 ± 2.31 | 4.28 ± 2.03 | NS |

**S2 Table. Summary statistics for Alzheimer’s Disease Neuroimaging Initiative (ADNI) participants with baseline cerebrospinal fluid (CSF) protein measurements**. Descriptive data is summarized by diagnostic category. Values represent the mean ± standard error, percent, or number of participants in a given diagnostic category. Two-tailed *P*-values were from analysis of variance (continuous traits) or chi-square (categorical values) tests by diagnostic group. CN – normal control; MCI – mild cognitive impairment; AD – Alzheimer’s disease; NS – not significant (P>0.05).
