# Supplementary material for: Fine-mapping of the human leukocyte antigen locus as a risk factor for Alzheimer disease: A case–control study
Source: PLoS Med. 2017 Mar 28;14(3):e1002272. doi: 10.1371/journal.pmed.1002272 (PMC5369701; doi:10.1371/journal.pmed.1002272)
Supplement: S3 Table — Analysis of patients with baseline AD diagnosis from the ADNI cohort carrying HLA A*03:01~B*07:02 (n = 3) versus haplotype noncarriers (n = 63) shows no significant differences in volumetric, clinical, cognitive, and biomarker assessments relevant to AD with the exception of amyloid β levels as measured in CSF (p = 0.03). The p-values for volumetric measurements are the effect of carrying the DR15 haplotype (binary 0/1) in a linear regression model adjusted for baseline age, sex, years of education, dose of APOE ε4 allele (0/1/2), and intracranial volume. The p-values for clinical and biomarker measures are the effect of carrying the DR15 haplotype (binary 0/1) in a linear regression model adjusted for baseline age, sex, years of education, and dose of APOE ε4 allele (0/1/2). ADAS11, 11-item Alzheimer’s Disease Assessment Scale cognitive subscale; MMSE, Mini-Mental State Exam. (DOCX) [file pmed.1002272.s011.docx]

**S3 Table: HLA-A*03:01~HLA-B*07:02 haplotype-carriers do not show any significant baseline differences on clinical biomarker measures of Alzheimer’s disease with the exception of CSF amyloid beta level.**

| **Measurement** | ***P*-value** |
| --- | --- |
| **Volumetrics** | |
| Whole brain volume | 0.34 |
| Ventricle volume | 0.86 |
| Middle temporal lobe volume | 0.93 |
| Hippocampus volume | 0.86 |
| Entorhinal cortex volume | 0.42 |
| Fusiform gyrus volume | 0.41 |
| **Clinical measures** | |
| CDR-SB score | 0.38 |
| MMSE score | 0.32 |
| ADAS11 score | 0.52 |
| RAVLT Forgetting score | 0.97 |
| **Biomarker measures** | |
| CSF amyloid beta level | 0.03* |
| CSF total tau level | 0.88 |
| CSF p-tau level | 0.74 |

**S3 Table: HLA-A*03:01~HLA-B*07:02 haplotype-carriers do not show any significant baseline differences on clinical biomarker measures of Alzheimer’s disease with the exception of CSF amyloid beta level.** Analysis of patients with baseline Alzheimer’s disease (AD) diagnosis from the ADNI cohort carrying HLA-A*03:01~HLA-B*07:02 (n=3) versus haplotype non-carriers (n=63) show no significant differences in volumetric, clinical, cognitive, and biomarker assessments relevant to AD with the exception of amyloid beta levels as measured in CSF (p=0.03, β±standard error = 47.19±21.78). P-value for volumetric measurements are the effect of carrying the DR15 haplotype (binary 0/1) in a linear regression model adjusted for baseline age, sex, years of education, dose of *APOE* ε4 allele (0/1/2), and intracranial volume. P-value for clinical and biomarker measures are the effect of carrying the DR15 haplotype (binary 0/1) in a linear regression model adjusted for baseline age, sex, years of education, and dose of *APOE* ε4 allele (0/1/2). CDR-SB – Clinical Dementia Rating Scale sum of boxes score; MMSE – Mini-Mental State Exam; ADAS11 – 11-item Alzheimer’s Disease Assessment Scale-Cognitive subscale; RAVLT – Rey Auditory Verbal Learning Test; CSF – cerebrospinal fluid.
