# Supplementary material for: Fine-mapping of the human leukocyte antigen locus as a risk factor for Alzheimer disease: A case–control study
Source: PLoS Med. 2017 Mar 28;14(3):e1002272. doi: 10.1371/journal.pmed.1002272 (PMC5369701; doi:10.1371/journal.pmed.1002272)
Supplement: S4 Table — Results from regression models used to determine the effect of dose of HLA risk haplotype DRB1*15:01~DQA1*01:02~DQB1*06:02 on longitudinal changes in ADAS and RAVLT cognitive test scores in cognitively normal, MCI, and AD groups from the ADNI cohort. The beta estimate (Estimate) and accompanying standard error (SE) reflect the adjusted effect of each independent variable as a predictor of ADAS 11-item score and RAVLT forgetting index scores. HLA haplotype dose demonstrated a significant positive and negative association with the rate of change in the ADAS (p = 0.02) and RAVLT (p = 0.03) scores, respectively, across all diagnostic groups (Time x Haplotype Dose). In other words, a greater dose of risk haplotype was associated with worse decline in cognitive performance over time. For all disease groups, the linear statistical model included the following as independent variables: age, time (from baseline), sex, CDR-SB score, APOE ε4 carrier status, education, and haplotype dose. All tests were two-tailed. (DOCX) [file pmed.1002272.s012.docx]

**S4 Table.**

| **Outcome** | **Variable** | **Estimate ± SE** | **P-Value** |
| --- | --- | --- | --- |
| ADAS  (11 Item) | Age | 0.02 ± 0.04 | 0.56 |
|  | Time | 5.59 ± 2.40 | 0.01 |
|  | Sex | -0.68 ± 0.49 | 0.16 |
|  | CDR-SB | 2.56 ± 0.12 | < 1x10^-6^ |
|  | Education | -0.16 ± 0.09 | 0.07 |
|  | *APOE*ε4 Status | 0.37 ± 0.36 | 0.31 |
|  | Haplotype Dose | -0.05 ± 0.47 | 0.92 |
|  | Time x Age | -0.07 ± 0.03 | 0.02 |
|  | Time x Sex | -0.07 ± 0.33 | 0.84 |
|  | Time x CDR-SB | 0.82 ± 0.09 | < 1x10^-6^ |
|  | Time x Education | -2.50 x 10^-3^ ± 0.06 | 0.97 |
|  | Time x *APOE* ε4 | 0.79 ± 0.25 | 1.20 x 10^-3^ |
|  | **Time x Haplotype Dose** | **0.69 ± 0.32** | **0.03** |
| RAVLT Forgetting Score | Age | 8.89 x 10^-3^ ± 0.02 | 0.6 |
|  | Time | 0.45 ± 0.50 | 0.36 |
|  | Sex | 0.25 ± 0.20 | 0.22 |
|  | CDR-SB | 0.14 ± 0.05 | 0.01 |
|  | Education | 5.65 x 10^-3^ ± 0.04 | 0.87 |
|  | *APOE*ε4 Status | 0.63 ± 0.15 | 1.00 x 10^-4^ |
|  | Haplotype Dose | 0.02 ± 0.20 | 0.91 |
|  | Time x Age | 9.70 x 10^-4^ ± 5.74 x 10^-3^ | 0.87 |
|  | Time x Sex | -0.08 ± 0.06 | 0.22 |
|  | Time x CDR-SB | -0.08 ± 0.03 | 2.80 x 10^-3^ |
|  | Time x Education | -0.02 ± 0.01 | 0.08 |
|  | Time x *APOE*ε4 | -0.10 ± 0.05 | 0.05 |
|  | **Time x Haplotype Dose** | **-0.15 ± 0.06** | **0.02** |

**S4 Table. Human Leukocyte Antigen (HLA) *DR15* risk haplotype dosage is associated with longitudinal changes in ADAS and RAVLT cognitive test scores**. Results from regression models used to determine the effect of dose of HLA risk haplotype DRB1*15:01~DQA*01:02~DQB1*06:02 on longitudinal changes in ADAS and RAVLT cognitive test scores in normal control, mild cognitive impairment, and Alzheimer’s disease groups from the Alzheimer’s Disease Neuroimaging Initiative cohort. The beta estimate (Estimate) and accompanying standard error (SE) reflect the adjusted effect of each independent variable as a predictor of Alzheimer’s Disease Assessment Scale (ADAS) 11 item score and Rey Auditory Verbal Learning Test (RAVLT) forgetting index scores. HLA haplotype dose demonstrated a significant positive and negative association with the rate of change in ADAS (p=0.02) and RAVLT (p=0.03) scores, respectively, across all diagnostic groups (Time x Haplotype Dose). In other words, greater dose of risk haplotype was associated with worse decline in cognitive performance over time. For all disease groups, the linear statistical model included as independent variables: age, time (from baseline), sex, Clinical Dementia Rating scale sum of boxes (CDR-SB) score, *APOE ε*4 carrier status, education, and haplotype dose. All tests were two-tailed.
