# Supplementary material for: Fine-mapping of the human leukocyte antigen locus as a risk factor for Alzheimer disease: A case–control study
Source: PLoS Med. 2017 Mar 28;14(3):e1002272. doi: 10.1371/journal.pmed.1002272 (PMC5369701; doi:10.1371/journal.pmed.1002272)
Supplement: S5 Table — Analysis of patients with baseline AD diagnosis from the ADNI cohort carrying DR15 (n = 23) versus DR15 noncarriers (n = 47) show no significant differences in volumetric, clinical, cognitive, and biomarker assessments relevant to AD. The p-values for volumetric measurements are the effect of carrying the DR15 haplotype (binary 0/1) in a linear regression model adjusted for baseline age, sex, years of education, dose of APOE ε4 allele (0/1/2), and intracranial volume. The p-values for clinical and biomarker measures are the effect of carrying the DR15 haplotype (binary 0/1) in a linear regression model adjusted for baseline age, sex, years of education, and dose of APOE ε4 allele (0/1/2). MMSE, Mini-Mental State Exam. (DOCX) [file pmed.1002272.s013.docx]

**S5 Table**

| **Measurement** | ***P*-value** |
| --- | --- |
| **Volumetrics** | |
| Whole brain volume | 0.53 |
| Ventricle volume | 0.93 |
| Middle temporal lobe volume | 0.66 |
| Hippocampus volume | 0.51 |
| Entorhinal cortex volume | 0.56 |
| Fusiform gyrus volume | 0.57 |
| **Clinical measures** | |
| CDR-SB score | 0.92 |
| MMSE score | 0.58 |
| ADAS11 score | 0.51 |
| RAVLT Forgetting score | 0.86 |
| **Biomarker measures** | |
| CSF amyloid beta level | 0.46 |
| CSF total tau level | 0.27 |
| CSF p-tau level | 0.30 |

**S5 Table: DR15 haplotype-carriers do not show any significant baseline differences on clinical biomarker measures of Alzheimer’s disease.** Analysis of patients with baseline Alzheimer’s disease (AD) diagnosis from the Alzheimer’s Disease Neuroimaging Initiative cohort carrying *DR15* (n=23) versus *DR15* non-carriers (n=47) show no significant differences in volumetric, clinical, cognitive, and biomarker assessments relevant to AD. P-value for volumetric measurements are the effect of carrying the *DR15* haplotype (binary 0/1) in a linear regression model adjusted for baseline age, sex, years of education, dose of *APOE* ε4 allele (0/1/2), and intracranial volume. P-value for clinical and biomarker measures are the effect of carrying the *DR15* haplotype (binary 0/1) in a linear regression model adjusted for baseline age, sex, years of education, and dose of *APOE* ε4 allele (0/1/2). CDR-SB – Clinical Dementia Rating Scale sum of boxes score; MMSE – Mini-Mental State Exam; ADAS11 – 11-item Alzheimer’s Disease Assessment Scale-Cognitive subscale; RAVLT – Rey Auditory Verbal Learning Test; CSF – cerebrospinal fluid.
