# Supplementary material for: Fine-mapping of the human leukocyte antigen locus as a risk factor for Alzheimer disease: A case–control study
Source: PLoS Med. 2017 Mar 28;14(3):e1002272. doi: 10.1371/journal.pmed.1002272 (PMC5369701; doi:10.1371/journal.pmed.1002272)
Supplement: S6 Table — Regression models were used to determine the effect of HLA risk haplotype DRB1*15:01~DQA1*01:02~DQB1*06:02 dose on cross-sectional CSF levels of chemokine CC-4 in the cognitively normal, MCI, and AD groups of the ADNI cohort are summarized. The beta estimate (Estimate) and accompanying standard error (SE) reflect the adjusted effect of each independent variable as a predictor of chemokine CC-4 levels. HLA risk haplotype dose demonstrated a significant positive association with baseline chemokine CC-4 CSF levels across all diagnostic groups (Haplotype Dose, p = 5.18 x 10^−3^) such that more copies of the risk haplotype were associated with higher levels of CSF chemokine CC-4, a measure of inflammation. For all disease groups, the linear statistical model included the following as independent variables: age, sex, CDR-SB score, APOE ε4 carrier status, education, and haplotype dose. All tests were two-tailed. (DOCX) [file pmed.1002272.s014.docx]

**S6 Table.**

| **Outcome** | **Variable** | **Estimate ± SE** | **P-Value** |
| --- | --- | --- | --- |
| Chemokine CC-4 | Age | 5.08 x 10^-3^ ± 2.23 x 10^-3^ | 0.02 |
|  | Sex | -0.15 ± 0.03 | 5.84 x 10^-7^ |
|  | CDR-SB | -8.29 x 10^-3^ ± 7.09 x 10^-3^ | 0.24 |
|  | Education | -9.8 x 10^-3^ ± 4.63 x 10^-3^ | 0.04 |
|  | *APOE*ε4 Status | -0.01 ± 0.02 | 0.53 |
|  | **Haplotype Dose** | **0.08 ± 0.03** | **5.18 x 10^-3^** |

**S6 Table.** **HLA *DR15* risk haplotype dosage is associated with baseline levels of Chemokine CC-4 in cerebrospinal fluid (CSF)**. Regression models were used to determine the effect of Human Leukocyte Antigen (HLA) risk haplotype DRB1*15:01~DQA*01:02~DQB1*06:02 dose on cross-sectional CSF levels of Chemokine CC-4 in the normal control, mild cognitive impairment, and Alzheimer’s disease groups of the Alzheimer’s Disease Neuroimaging Initiative cohort are summarized. The beta estimate (Estimate) and accompanying standard error (SE) reflect the adjusted effect of each independent variable as a predictor of Chemokine CC-4 levels. HLA risk haplotype dose demonstrated a significant positive association with baseline Chemokine CC-4 CSF levels across all diagnostic groups (Haplotype Dose, p=5.18 x 10^-3^) such that more copies of the risk haplotype were associated with higher levels of CSF Chemokine CC-4, a measure of inflammation. For all disease groups, the linear statistical model included as independent variables: age, sex, Clinical Dementia Rating scale sum of boxes (CDR-SB) score, *APOE ε*4 carrier status, education, and haplotype dose. All tests were two-tailed.
