# Supplementary material for: Fine-mapping of the human leukocyte antigen locus as a risk factor for Alzheimer disease: A case–control study
Source: PLoS Med. 2017 Mar 28;14(3):e1002272. doi: 10.1371/journal.pmed.1002272 (PMC5369701; doi:10.1371/journal.pmed.1002272)
Supplement: S7 Table — All significant five-allele haplotype results (*p < 0.05) for the combined UCSF + ADGC cohort (n = 11,690) when males and females are analyzed separately. Two of the three most significant five-allele haplotypes from the combined analysis (males + females) were significant in an individual sex analysis and are highlighted in this table in bold. Nonsignificant results are shown in grey. In addition to the OR with the 95% CI, a breakdown of haplotype frequency in individuals with AD versus cognitively normal older adult controls is also provided. (DOCX) [file pmed.1002272.s015.docx]

**S7 Table**

|  | **Men** | | | | **Women** | | | |
| --- | --- | --- | --- | --- | --- | --- | --- | --- |
|  | **n = 4860 (2369 cases, 2491 controls)** | | | | **n = 6930 (3402 cases, 3428 controls)** | | | |
| **Significant** **Haplotypes** |  |  | **Frequency** | |  |  | **Frequency** | |
| ***A~B~DRB1~DQA1~DQB1*** | **OR (95% CI)** | ***P*-val** | **Controls** | **Cases** | **OR (95% CI)** | ***P*-val** | **Controls** | **Cases** |
| 01:01~37:01~15:01~01:02~06:02 | 0.33 (0.11 - 0.86) | 0.01* | 0.004 | 0.001 | 1.27 (0.67 - 2.46) | 0.43 | 0.003 | 0.004 |
| 02:01~07:02~01:01~01:01~05:01 | 1.42 (0.73 - 2.84) | 0.27 | 0.003 | 0.005 | 2.52 (1.06 - 6.63) | 0.02* | 0.001 | 0.003 |
| **02:01~13:02~07:01~02:01~02:02** | **0.68 (0.42 - 1.09)** | **0.09** | **0.010** | **0.007** | **0.68 (0.46 - 0.99)** | **0.03*** | **0.011** | **0.007** |
| 02:01~15:01~07:01~02:01~02:02 | n/a | n/a | 0.001 | 0.001 | 0.25 (0.05 - 0.93) | 0.02* | 0.002 | 0.000 |
| 02:01~27:05~08:01~04:01~04:02 | 1.05 (0.37 - 2.99) | 0.92 | 0.002 | 0.002 | 0.3 (0.09 - 0.84) | 0.01* | 0.002 | 0.001 |
| 02:01~40:01~04:01~03:01~03:02 | 0.53 (0.12 - 1.96) | 0.29 | 0.002 | 0.001 | 2.74 (1.10 - 7.72) | 0.02* | 0.001 | 0.003 |
| 02:01~44:02~03:01~05:01~02:01 | 5.79 (1.26 - 53.79) | 0.01* | 0.000 | 0.002 | 1.16 (0.52 - 2.66) | 0.69 | 0.002 | 0.002 |
| 02:01~44:02~13:01~01:03~06:03 | 1.71 (1.00 - 2.99) | 0.04* | 0.005 | 0.008 | 1.38 (0.89 - 2.16) | 0.13 | 0.006 | 0.008 |
| 02:01~52:01~15:02~01:03~06:01 | n/a | n/a | 0.000 | 0.000 | 5.04 (1.07 - 47.36) | 0.02* | 0.000 | 0.001 |
| 02:01~57:01~07:01~02:01~03:03 | 1.81 (1.17 - 2.84) | 4.74*10^-3^* | 0.007 | 0.013 | 1.10 (0.08 - 1.52) | 0.55 | 0.011 | 0.012 |
| **03:01~07:02~15:01~01:02~06:02** | **1.31 (1.09 - 1.58)** | **3.46*10^-3^*** | **0.045** | **0.058** | **1.12 (0.96 - 1.30)** | **0.16** | **0.049** | **0.055** |
| 03:01~15:01~13:01~01:03~06:03 | 0.28 (0.07 - 0.88) | 0.02* | 0.003 | 0.001 | 1.21 (0.48 - 3.13) | 0.66 | 0.001 | 0.002 |
| 03:01~27:05~01:01~01:01~05:01 | n/a | n/a | 0.000 | 0.001 | 0.07 (0 - 0.47) | 8.27*10^-4^* | 0.002 | 0.000 |
| 03:01~57:01~07:01~02:01~03:03 | 0.42 (0.16 - 1.00) | 0.03* | 0.004 | 0.002 | 1.73 (0.63 - 5.19) | 0.24 | 0.001 | 0.002 |
| 11:01~35:01~07:01~02:01~02:02 | n/a | n/a | 0.000 | 0.000 | 0.15 (0.02 - 0.68) | 4.70*10^-3^* | 0.002 | 0.000 |
| 11:01~51:01~01:01~01:01~05:01 | n/a | n/a | 0.000 | 0.000 | 0.22 (0.02 - 1.08) | 0.04* | 0.001 | 0.000 |
| 26:01~27:05~01:01~01:01~05:01 | 3.42 (1.06 - 14.42) | 0.02* | 0.001 | 0.003 | 0.76 (0.28 - 1.95) | 0.52 | 0.002 | 0.001 |
| 68:01~44:02~11:01~05:05~03:01 | 0.42 (0.16 - 1.00) | 0.03* | 0.004 | 0.002 | 1.76 (0.69 - 4.86) | 0.19 | 0.001 | 0.002 |
|  |  |  |  |  |  |  |  |  |

**S7 Table. 5-allele haplotypes with significant risk associations in individual sexes.** All significant 5-allele haplotype results (*p<0.05) for combined University of California, San Francisco + Alzheimer’s Disease Genetics Consortium cohort (n = 11,690) when males and females are analyzed separately. Two of the three most significant 5-allele haplotypes from the combined analysis (males + females) were significant in an individual sex analysis and are highlighted in this table in bold. Non-significant results are shown in grey. In addition to odds ratio (OR) with 95% confidence interval (CI), a breakdown of haplotype frequency in Alzheimer’s disease cases versus healthy older adult controls is also provided.
