# Supplementary material for: Fine-mapping of the human leukocyte antigen locus as a risk factor for Alzheimer disease: A case–control study
Source: PLoS Med. 2017 Mar 28;14(3):e1002272. doi: 10.1371/journal.pmed.1002272 (PMC5369701; doi:10.1371/journal.pmed.1002272)
Supplement: S8 Table — All significant class I (two-allele) and class II (three-allele) haplotype results (*p < 0.05) for the combined UCSF + ADGC cohort (n = 11,690) when males and females are analyzed separately. Class I and class II haplotypes present in one of the three most significant five-allele from the combined analysis (males + females) are highlighted in this table in bold. Nonsignificant results are shown in grey. In addition to OR with 95% CI, a breakdown of haplotype frequency in individuals with AD versus cognitively normal older adult controls is also provided. (DOCX) [file pmed.1002272.s016.docx]

**S8 Table.**

|  | **Males** | | | | **Females** | | | |
| --- | --- | --- | --- | --- | --- | --- | --- | --- |
|  | **n = 4860 (2369 cases, 2491 controls)** | | | | **n = 6930 (3402 cases, 3428 controls)** | | | |
| **Class I** |  |  | **Frequency** | |  |  | **Frequency** | |
| ***A~B*** | **OR (95% CI)** | ***P*-val** | **Controls** | **Cases** | **OR (95% CI)** | ***P*-val** | **Controls** | **Cases** |
| **02:01~13:02** | **0.67 (0.43 - 1.03)** | **0.05** | **0.012** | **0.008** | **0.63 (0.45 - 0.88)** | **4.89*10^-3*^** | **0.014** | **0.009** |
| 02:01~18:01 | 1.23 (0.77 - 2.00) | 0.36 | 0.007 | 0.009 | 1.53 (1.03 - 2.28) | 0.03* | 0.007 | 0.010 |
| 02:01~55:01 | 0.53 (0.14 - 1.69) | 0.23 | 0.002 | 0.001 | 5.04 (1.07 - 47.36) | 0.02* | 0.000 | 0.001 |
| **03:01~07:02** | **1.19 (1.02 - 1.39)** | **0.03*** | **0.069** | **0.080** | **1.06 (0.93 - 1.20)** | **0.40** | **0.072** | **0.075** |
| 03:01~15:01 | 0.44 (0.25 - 0.75) | 1.18*10^-3*^ | 0.010 | 0.004 | 0.88 (0.56 - 1.37) | 0.54 | 0.007 | 0.006 |
| 03:01~27:05 | 0.89 (0.44 - 1.80) | 0.73 | 0.004 | 0.004 | 0.50 (0.24 - 1.02) | 0.04* | 0.004 | 0.002 |
| 03:01~40:01 | 1.90 (0.83 - 4.60) | 0.10 | 0.002 | 0.004 | 0.43 (0.20 - 0.88) | 0.01* | 0.004 | 0.002 |
| 03:01~55:01 | 1.40 (0.43 - 4.91) | 0.53 | 0.001 | 0.002 | 4.54 (0.94 - 43.15) | 0.03* | 0.000 | 0.001 |
| 11:01~15:01 | 1.05 (0.28 - 3.94) | 0.93 | 0.001 | 0.001 | 0.44 (0.20 - 0.93) | 0.02* | 0.004 | 0.002 |
| 11:01~44:02 | 0.83 (0.43 - 1.59) | 0.55 | 0.005 | 0.004 | 0.46 (0.20 - 0.98) | 0.03* | 0.004 | 0.002 |
| 11:01~44:03 | n/a | n/a | 0.001 | 0.001 | 2.69 (1.00 - 8.40) | 0.03* | 0.001 | 0.002 |
| **24:02~38:01** | **0.10 (0 - 0.66)** | **0.01*** | **0.002** | **0.000** | 0.67 (0.20 - 2.11) | 0.45 | 0.001 | 0.001 |
| 68:01~40:01 | 0.43 (0.17 - 0.97) | 0.03* | 0.004 | 0.002 | 0.48 (0.26 - 0.86) | 0.01* | 0.006 | 0.003 |
|  |  |  |  |  |  |  |  |  |
| **Class II** |  |  | **Frequency** | |  |  | **Frequency** | |
| ***DRB1~DQB1~DQA1*** | **OR (95% CI)** | ***P*-val** | **Controls** | **Cases** | **OR (95% CI)** | ***P*-val** | **Controls** | **Cases** |
| 10:01~01:05~05:01 | 2.21 (1.00 - 5.27) | 0.03* | 0.002 | 0.004 | 0.88 (0.47 - 1.65) | 0.67 | 0.004 | 0.003 |
| 11:01~05:05~03:01 | 0.66 (0.46 - 0.94) | 0.02* | 0.017 | 0.011 | 1.01 (0.75 - 1.35) | 0.96 | 0.014 | 0.014 |
| 12:01~05:05~03:01 | 1.05 (0.77 - 1.45) | 0.74 | 0.017 | 0.018 | 0.72 (0.55 - 0.94) | 0.01* | 0.020 | 0.015 |
| 13:02~01:02~06:09 | 1.03 (0.70 - 1.53) | 0.86 | 0.011 | 0.012 | 0.69 (0.47 - 0.99) | 0.04* | 0.011 | 0.008 |
| **15:01~01:02~06:02** | **1.14 (1.03 - 1.27)** | **0.01*** | **0.173** | **0.193** | **1.04 (0.95 - 1.13)** | **0.43** | **0.182** | **0.187** |

**S8 Table. Class I and class II haplotypes with significant risk associations in individual sexes.** All significant class I (2-allele) and class II (3-allele) haplotype results (*p<0.05) for combined University of California, San Francisco + Alzheimer’s Disease Genetics Consortium cohort (n = 11,690) when males and females are analyzed separately. Class I and class II haplotypes present in one of the three most significant 5-allele from the combined analysis (males + females) are highlighted in this table in bold. Non-significant results are shown in grey. In addition to odds ratio (OR) with 95% confidence interval (CI), a breakdown of haplotype frequency in Alzheimer’s disease cases versus healthy older adult controls is also provided.
