# Supplementary material for: Fine-mapping of the human leukocyte antigen locus as a risk factor for Alzheimer disease: A case–control study
Source: PLoS Med. 2017 Mar 28;14(3):e1002272. doi: 10.1371/journal.pmed.1002272 (PMC5369701; doi:10.1371/journal.pmed.1002272)
Supplement: S9 Table — All significant loci results (*p < 0.05) for combined UCSF + ADGC cohort (n = 11,690) when males and females are analyzed separately. Alleles present in one of the top three most significant five-allele haplotypes from the combined analysis (A*02:01~B*13:02~DRB1*07:01~DQA1*02:01~DQB1*02:02, A*03:01~B*07:02~DRB1*15:01~DQA1*01:02~DQB1*06:02 and A*24:02~B*38:01~DRB1*13:01~DQA1*01:03~DQB1*06:03) are shown in this table in bold. Nonsignificant results are shown in grey. In addition to OR with 95% CI, a breakdown of allele frequency in individuals with AD versus cognitively normal older adult controls is also provided. (DOCX) [file pmed.1002272.s017.docx]

**S9 Table.**

|  | **Males** | | | | **Females** | | | |
| --- | --- | --- | --- | --- | --- | --- | --- | --- |
|  | **n = 4860 (2369 cases, 2491 controls)** | | | | **n = 6930 (3402 cases, 3428 controls)** | | | |
|  |  |  | **Frequency** | |  |  | **Frequency** | |
| **Significant Alleles:** | **OR (95% CI)** | ***P*-val** | **Controls** | **Cases** | **OR (95% CI)** | ***P-*val** | **Controls** | **Cases** |
| A*23:01 | 0.91 (0.68 - 1.22) | 0.51 | 0.0209 | 0.0190 | 0.75 (0.59 - 0.96) | 0.02* | 0.0233 | 0.0176 |
| A*32:01 | 1.32 (1.05 - 1.67) | 0.02* | 0.0279 | 0.0365 | 0.96 (0.80 - 1.16) | 0.70 | 0.0349 | 0.0337 |
| **B 07:02** | **1.14 (1.03 - 1.27)** | **0.01*** | **0.1626** | **0.1815** | **1.03 (0.94 - 1.12)** | **0.59** | **0.1631** | **0.1665** |
| B 15:01 | 0.83 (0.70 - 0.99) | 0.03* | 0.0634 | 0.0532 | 0.91 (0.79 - 1.05) | 0.18 | 0.0621 | 0.0567 |
| B 39:01 | 1.09 (0.64 - 1.86) | 0.75 | 0.0060 | 0.0065 | 0.58 (0.36 - 0.91) | 0.01* | 0.0079 | 0.0046 |
| B 58:01 | 2.11 (1.12 - 4.12) | 0.01* | 0.0032 | 0.0068 | 0.78 (0.45 - 1.32) | 0.32 | 0.0051 | 0.0040 |
| DRB1 10:01 | 2.21 (1.00 - 5.27) | 0.03* | 0.0020 | 0.0044 | 0.88 (0.47 - 1.65) | 0.67 | 0.0035 | 0.0031 |
| DRB1 11:01 | 0.66 (0.46 - 0.94) | 0.02* | 0.0173 | 0.0114 | 1.01 (0.75 - 1.35) | 0.96 | 0.0142 | 0.0143 |
| DRB1 12:01 | 1.05 (0.77 - 1.45) | 0.74 | 0.0169 | 0.0177 | 0.72 (0.55 - 0.94) | 0.01* | 0.0203 | 0.0147 |
| **DRB1 15:01** | **1.15 (1.03 - 1.27)** | **9.60*10^-3^*** | **0.1744** | **0.1948** | **1.03 (0.94 - 1.13)** | **0.49** | **0.1832** | **0.1878** |
| **DQA1 01:02** | **1.11 (1.01 - 1.22)** | **0.03*** | **0.2355** | **0.2545** | **1.03 (0.96 - 1.12)** | **0.41** | **0.2386** | **0.2447** |
| DQA1 01:05 | 2.21 (1.00 - 5.27) | 0.03* | 0.0020 | 0.0044 | 0.88 (0.47 - 1.65) | 0.67 | 0.0035 | 0.0031 |
| **DQB1 06:02** | **1.14 (1.03 - 1.27)** | **0.01*** | **0.1728** | **0.1929** | **1.03 (0.95 - 1.13)** | **0.45** | **0.1820** | **0.1871** |
| DQB1 06:09 | 1.03 (0.70 - 1.53) | 0.86 | 0.0112 | 0.0116 | 0.69 (0.47 - 0.99) | 0.04* | 0.0111 | 0.0076 |

**S9 Table. Individual alleles with significant risk associations in individual sexes.** All significant loci results (*p<0.05) for combined University of California, San Francisco + Alzheimer’s Disease Genetics Consortium cohort (n = 11,690) when males and females are analyzed separately. Alleles present in one of the top three most significant 5-allele haplotypes (02:01~13:02~07:01~02:01~02:02, 03:01~07:02~15:01~01:02~06:02 and 24:02~38:01~13:01~01:03~06:03) are shown in this table in bold. Non-significant results are shown in grey. In addition to odds ratio (OR) with 95% confidence interval (CI), a breakdown of allele frequency in Alzheimer’s disease cases versus healthy older adult controls is also provided.
