# Supplementary material for: Fine-mapping of the human leukocyte antigen locus as a risk factor for Alzheimer disease: A case–control study
Source: PLoS Med. 2017 Mar 28;14(3):e1002272. doi: 10.1371/journal.pmed.1002272 (PMC5369701; doi:10.1371/journal.pmed.1002272)
Supplement: S10 Table — We randomly split our full ADGC + UCSF cohort (n = 11,381) in half ten times, balancing AD and cognitively normal controls, to determine how often the haplotypes we found to be significant (p < 0.05) in our original analysis replicated in these 20 smaller cohort analyses. The OR, 95% CI, and p-value from the original analysis (values from Table 2) are listed for each haplotype, and results are listed for any of the replication cohorts in which a given haplotype was significant. Each haplotype also lists the number of smaller cohorts in which a significant finding was found (n rep) and the percentage (number of replication cohorts / 20). In these 20 analyses, two of our top-associated five-allele haplotypes, A*03:01~B*07:02~DRB1*15:01~DQA1*01:02~DQB1*06:02 and A*02:01~B*13:02~DRB1*07:01~DQA1*02:01~DQB1*02:02, showed up as significant in over half of the smaller analyses, further corroborating their role in AD risk. (DOCX) [file pmed.1002272.s018.docx]

**S10 Table.**

|  | **A~B~DRB1~DQA1~DQB1** | **OR (95% CI)** | ***P-*value** | **n rep:** | **%** |
| --- | --- | --- | --- | --- | --- |
| **Original cohort:** | **01:01~08:01~07:01~02:01~03:03** | **0.43 (0.16 - 1.02)** | **0.037** | 5 | 25 |
| Replication cohorts: | A.1 | 0.09 (0 - 0.63) | 0.004 |  |  |
|  | B.2 | 0.34 (0.08 - 1.11) | 0.048 |  |  |
|  | C.2 | 0.22 (0.02 - 1.09) | 0.037 |  |  |
|  | I.2 | 0.22 (0.02 - 1.09) | 0.037 |  |  |
|  | J.1 | 0.34 (0.08 - 1.12) | 0.049 |  |  |
| **Original cohort:** | **02:01~13:02~07:01~02:01~02:02** | **0.66 (0.5 - 0.89)** | **0.004** | 11 | 55 |
| Replication cohorts: | A.2 | 0.55 (0.34 - 0.86) | 0.006 |  |  |
|  | B.1 | 0.50 (0.31 - 0.77) | 0.001 |  |  |
|  | C.1 | 0.66 (0.45 - 0.98) | 0.030 |  |  |
|  | D.2 | 0.57 (0.35 - 0.89) | 0.009 |  |  |
|  | E.1 | 0.55 (0.35 - 0.85) | 0.005 |  |  |
|  | F.2 | 0.47 (0.31 - 0.71) | 0.0001 |  |  |
|  | G.1 | 0.62 (0.39 - 0.99) | 0.033 |  |  |
|  | G.2 | 0.65 (0.44 - 0.96) | 0.022 |  |  |
|  | H.2 | 0.64 (0.42 - 0.97) | 0.027 |  |  |
|  | I.1 | 0.61 (0.39 - 0.93) | 0.017 |  |  |
|  | J.1 | 0.54 (0.34 - 0.83) | 0.003 |  |  |
| **Original cohort:** | **02:01~15:01~07:01~02:01~02:02** | **0.39 (0.14 - 0.99)** | **0.030** | 6 | 30 |
| Replication cohorts: | A.1 | 0.20 (0.04 - 0.72) | 0.005 |  |  |
|  | C.1 | 0.22 (0.02 - 1.09) | 0.037 |  |  |
|  | D.1 | 0.22 (0.04 - 0.78) | 0.008 |  |  |
|  | E.2 | 0.18 (0.02 - 0.84) | 0.013 |  |  |
|  | H.1 | 0.08 (0 - 0.57) | 0.002 |  |  |
|  | I.2 | 0.28 (0.05 - 1.05) | 0.034 |  |  |
| **Original cohort:** | **02:01~44:02~13:01~01:03~06:03** | **1.44 (1.03 - 2.03)** | **0.027** | 6 | 30 |
| Replication cohorts: | A.2 | 2.17 (1.28 - 3.79) | 0.002 |  |  |
|  | D.2 | 1.54 (0.98 - 2.44) | 0.047 |  |  |
|  | E.1 | 1.69 (1.03 - 2.84) | 0.029 |  |  |
|  | G.2 | 2.55 (1.49 - 4.52) | 0.0003 |  |  |
|  | H.1 | 1.59 (0.98 - 2.61) | 0.045 |  |  |
|  | I.2 | 1.71 (1.07 - 2.76) | 0.018 |  |  |
| **Original cohort:** | **02:01~57:01~07:01~02:01~03:03** | **1.31 (1.01 - 1.69)** | **0.038** | 6 | 30 |
| Replication cohorts: | B.1 | 1.71 (1.15 - 2.55) | 0.005 |  |  |
|  | C.2 | 1.69 (1.16 - 2.49) | 0.004 |  |  |
|  | D.2 | 1.79 (1.22 - 2.65) | 0.002 |  |  |
|  | F.1 | 1.51 (1.02 - 2.25) | 0.031 |  |  |
|  | G.2 | 1.47 (1.02 - 2.12) | 0.030 |  |  |
|  | H.1 | 1.65 (1.14 - 2.4) | 0.005 |  |  |
| **Original cohort:** | **03:01~07:02~12:01~05:05~03:01** | **0.30 (0.09 - 0.84)** | 0.011 | 1 | 5 |
| Replication cohorts: | H.2 | 0.34 (0.08 - 1.11) | 0.048 |  |  |
| **Original cohort:** | **03:01~07:02~15:01~01:02~06:02** | **1.22 (1.08 - 1.38)** | **0.001** | 11 | 55 |
| Replication cohorts: | A.1 | 1.24 (1.05 - 1.46) | 0.009 |  |  |
|  | A.2 | 1.21 (1.02 - 1.44) | 0.026 |  |  |
|  | B.2 | 1.34 (1.14 - 1.59) | 0.0004 |  |  |
|  | C.1 | 1.26 (1.07 - 1.49) | 0.006 |  |  |
|  | D.1 | 1.40 (1.18 - 1.65) | 0.0001 |  |  |
|  | E.2 | 1.22 (1.03 - 1.44) | 0.017 |  |  |
|  | F.2 | 1.26 (1.06 - 1.5) | 0.007 |  |  |
|  | G.1 | 1.26 (1.07 - 1.49) | 0.005 |  |  |
|  | H.1 | 1.30 (1.10 - 1.55) | 0.002 |  |  |
|  | I.2 | 1.35 (1.14 - 1.61) | 0.0004 |  |  |
|  | J.2 | 1.35 (1.14 - 1.6) | 0.0004 |  |  |
| **Original cohort:** | 11:01~35:01~07:01~02:01~02:02 | 0.31 (0.07 - 1.01) | 0.031 | 0 | 0 |
| Replication cohorts: | none |  |  |  |  |
| **Original cohort:** | **24:02~38:01~13:01~01:03~06:03** | **0.14 (0.02 - 0.63)** | **0.003** | 4 | 20 |
| Replication cohorts: | A.2 | 0.10 (0 - 0.71) | 0.007 |  |  |
|  | B.1 | 0.22 (0.02 - 1.09) | 0.037 |  |  |
|  | E.1 | 0.22 (0.02 - 1.09) | 0.037 |  |  |
|  | H.2 | 0.08 (0 - 0.52) | 0.001 |  |  |
| **Original cohort:** | 24:02~44:05~01:01~01:01~05:01 | 4.56 (0.94 - 43.38) | 0.033 | 0 | 0 |
| Replication cohorts: | none |  |  |  |  |
| **Original cohort:** | 29:02~58:01~08:04~04:01~04:02 | 4.56 (0.94 - 43.38) | 0.033 | 0 | 0 |
| Replication cohorts: | none |  |  |  |  |
| **Original cohort:** | **68:01~44:02~01:01~01:01~05:01** | **1.96 (0.99 - 4.04)** | **0.038** | 7 | 35 |
| Replication cohorts: | A.2 | 2.84 (0.97 - 10.09) | 0.036 |  |  |
|  | B.1 | 2.84 (0.97 - 10.08) | 0.036 |  |  |
|  | C.2 | 3.55 (1.11 - 14.83) | 0.017 |  |  |
|  | E.1 | 3.55 (1.11 - 14.82) | 0.017 |  |  |
|  | F.2 | 3.04 (1.15 - 9.35) | 0.013 |  |  |
|  | G.2 | 3.05 (1.16 - 9.38) | 0.013 |  |  |
|  | I.2 | 3.25 (1.14 - 11.35) | 0.015 |  |  |

**S10 Table. Twenty iterative analyses of randomly-split Alzheimer’s Disease Genetics Consortium (ADGC) + University of California, San Francisco (UCSF) combined cohort to corroborate top 5-allele haplotype associations with Alzheimer’s disease risk.** We randomly split our full ADGC + UCSF cohort (n = 11,381) in half 10 times, balancing cases and controls, to determine how often the haplotypes we found to be significant (p < 0.05) in our original analysis replicated in these 20 smaller cohort analyses. Odds ratio (OR), 95% confidence interval (CI), and p-value from original analysis (values from Table 2) are listed for each haplotype, and results are listed for any of the replication cohorts in which a given haplotype was significant. Each haplotype also lists the number of smaller cohorts in which a significant finding was found (n rep) and the percentage (number of replication cohorts / 20). In these 20 analyses, two of our top-associated 5-allele haplotypes, 03:01~07:02~15:01~01:02~06:02 and 02:01~13:02~07:01~02:01~02:02 showed up as significant in over half of the smaller analyses, further corroborating their role in Alzheimer’s disease risk.
